# Genome-Wide Characterization of the C-repeat Binding Factor (CBF) Gene Family Involved in the Response to Abiotic Stresses in Tea Plant (*Camellia sinensis*)

**DOI:** 10.3389/fpls.2020.00921

**Published:** 2020-07-23

**Authors:** Zhe Hu, Qiuyan Ban, Jing Hao, Xiangxiang Zhu, Yaohua Cheng, Junlin Mao, Mengling Lin, Enhua Xia, Yeyun Li

**Affiliations:** State Key Laboratory of Tea Plant Biology and Utilization, International Joint Laboratory on Tea Chemistry and Health Effects, Anhui Agricultural University, Hefei, China

**Keywords:** *Camellia sinensis*, *CBF* gene family, cold stress, functional genomics, transgenic *Arabidopsis thaliana*

## Abstract

C-repeat (CRT)/dehydration responsive element (DRE)-binding factor CBFs, a small family of genes encoding transcriptional activators, play important roles in plant cold tolerance. In this study, a comprehensive genome-wide analysis was carried out to identify and characterize the functional dynamics of *CsCBFs* in tea plant (*Camellia sinensis*). A total of 6 *CBF* genes were obtained from the tea plant genome and named *CBF1-6*. All of the *CsCBFs* had an AP2/ERF DNA-binding domain and nuclear localization signal (NLS) sequence. CsCBF-eGFP fusion and DAPI staining analysis confirmed the nuclear localization of the CsCBFs. Transactivation assays showed that the CsCBFs, except CsCBF1, had transcriptional activity. CsCBF expression was differentially induced by cold, heat, PEG, salinity, ABA, GA, MeJA, and SA stresses. In particular, the *CsCBF* genes were significantly induced by cold treatments. To further characterize the functions of *CsCBF* genes, we overexpressed the *CsCBF3* gene in *Arabidopsis thaliana* plants. The resulting transgenic plants showed increased cold tolerance compared with the wild-type *Arabidopsis* plant. The enhanced cold tolerance of the transgenic plants was potentially achieved through an ABA-independent pathway. This study will help to increase our understanding of *CsCBF* genes and their contributions to stress tolerance in tea plants.

## Introduction

Abiotic stresses in the natural environment, including low temperature, drought, and high salinity, seriously affect the growth, development, distribution, and productivity of plants (Kulik et al., 2011). Plants have evolved a complex set of adaptive mechanisms to survive these adverse environmental conditions, and these mechanisms involve a number of biochemical and physiological changes (Ramanjulu and Bartels, 2002). Different studies have suggested that transcription factors can activate the expression of related genes to protect plants from adversity (Agarwal and Jha, 2010). CBFs/DREBs (C-repeat binding factors/dehydration responsive element-binding factors) belong to the AP2/ERF (APETALA2/ethylene-responsive element-binding factor) transcription factor family (Riechmann and Meyerowitz, 1998) and have been reported to play pivotal roles in freezing tolerance and cold acclimation (Stockinger et al., 1997). The AP2/ERF family contains a highly conserved AP2/ERF domain that harbors a DNA-binding motif of ~60 amino acids (Sakuma et al., 2002). When plants suffer from low temperatures, related regulatory proteins are modified to regulate the expression of the *CBF* gene. For example, the inducers of CBF expression 1 (ICE1), calmodulin-binding transcription activator 3 (CAMTA3) and brassinazole-resistant 1 (BZR1), positively regulate CBF expression, whereas MYB15, ethylene-insensitive 3 (EIN3), and 14-3-3 negatively regulate CBF expression (Liu et al., 2017). Subsequently, CBF/DREB1 proteins bind to the cold- and dehydration-responsive DNA regulatory element designated the CRT (C-repeat)/DRE (dehydration response element), which is present in the promoters of COR (cold-regulated) genes and contains the core motif of G/ACCGAC (Yamaguchi-Shinozaki and Shinozaki, 1994; Huang et al., 2012), and to the promoters of other cold responsive genes, such as *COR15A, RD29A*, and *COR78* (Liu et al., 1998; Lucas et al., 2011; Akhtar et al., 2012), and stimulates their transcription (Baker et al., 1994; Jaglo et al., 2001; Zhao T. et al., 2012). Previous studies have identified a number of *CBF/DREB1 genes* and verified their functions in *Arabidopsis thaliana* (Novillo et al., 2007) and other plant species, such as cotton (*Gossypium hirsutum*) (Shan et al., 2007), wheat (*Triticum aestivum*) (Shen et al., 2003), rice (*Oryza sativa*) (Wang et al., 2008), maize (*Zea mays*) (Qin et al., 2004), soybean (*Glycine max*) (Kidokoro et al., 2015), and tomato (*Lycopersicon esculentum*) (Zhang et al., 2004). These findings suggested the conserved roles of *CBF/DREB1 genes* in the regulation of freezing tolerance across diverse plant species. Nevertheless, CBFs play different roles in responses to stress in different plant species (Ebrahimi et al., 2015). In addition to the cold response, *CBFs* could also respond to other abiotic stresses and hormones, such as heat, drought, salt, and abscisic acid (ABA) (Dubouzet et al., 2003; Xiao et al., 2006; Nada and Abogadallah, 2015).

Tea plants are the world's most important nonalcoholic beverage crop with a wealth of health benefits (Mukhopadhyay et al., 2016). The growth of tea plants is seriously affected by abiotic stresses (Wang et al., 2014), particularly extreme temperature and drought (Das et al., 2016; Liu et al., 2016; Hou et al., 2018; Zhou et al., 2018). Although several recent studies have demonstrated that *CsCBF1* enables cold stress in tea plants (Wang et al., 2012), overexpression of *CsDREB* increases salt and drought tolerance in transgenic *Arabidopsis thaliana* (Wang et al., 2017). However, given that the CBF transcription factor is a polygenic family, it is still unclear whether there are other new CBF genes in tea plants that respond to low temperature. In the present study, we thoroughly investigated the *CBF* genes in tea plant using tea plant genome and transcriptome datasets, with the aim of providing novel insights into the functional dynamics of CBF genes in tea plant. The overall obtained results provide a foundation for additional studies of the biological functions of *CsCBFs* under abiotic stresses and a new perspective for resistance breeding in tea plants.

## Materials and Methods

### Plant Materials and Stress Treatments

One-year-old tea cutting seedlings of the Shuchazao cultivar were planted in a pot and grown with a natural photoperiod in a greenhouse (12 h light and 12 h dark photoperiod, 25°C temperature and 70% relative humidity) (Li et al., 2016) at the State Key Laboratory of Tea Plant Biology and Utilization, Anhui Agricultural University (Hefei, China). For low and high temperature stresses, tea plants were grown at 4 and 38°C in a plant growth chamber. For the GA, ABA, SA, and MeJA treatments, a working solution of 100 μM was foliar sprayed on plants (Pan et al., 2017). For drought and salinity, the plants were transferred to a 20% PEG6000 and 200 mM NaCl solution (Li et al., 2010; Yue et al., 2014). The second or third mature leaves were harvested for gene analysis 0, 4, 12, and 24 h (Agarwal M. et al., 2006) after treatments. After collection, the samples were immediately frozen in liquid nitrogen and stored at −80°C for RNA extraction. Three biological replicates were conducted.

### Identification of CsCBF Genes and Phylogenetic Construction

The protein sequences of AtCBFs were downloaded from the NCBI (https://www.ncbi.nlm.nih.gov/) database and used as queries to search homologous sequences against the published genomes of tea plant (Wei et al., 2018; Xia E. et al., 2019) with the BlastP program at an *e*-value of 10^−3^ (Pan et al., 2017). The biophysical properties of the CsCBFs were computed using the online ProtParam tool (https://web.expasy.org/protparam/). The protein sequences of *Camellia sinensis* and *Arabidopsis thaliana* were aligned by DNAMAN6.0. Multiple sequence alignments of *Camellia sinensis* and *Arabidopsis thaliana* were analyzed using MEGA 6.0 (http://www.megasoftware.net/). The phylogenetic tree was constructed using the neighbor-joining algorithm with 1,000 replicates.

### Characterization of CsCBF Genes and Proteins

The promoter sequences ~2 kb upstream of the transcription start site of each *CsCBF* gene were identified, and the cis-elements were analyzed by the online tool Plant CARE (http://bioinformatics.psb.ugent.be/webtools/plantcare/html/). The full-length amino acid sequences of CsCBF were entered into the MEME (http://meme-suite.org/tools/meme) analysis tool to find their conserved motifs. Parameters of MEME are following: number of different motifs: 10, Minimum/Maximum motif width: 6/100.

### Subcellular Localization of CsCBFs

The full-length ORFs of *CsCBF* sequences were cloned into the pK7WGF2.0 vector containing the enhanced green fluorescent protein (eGFP) reporter gene by the gateway method. The isolated DNA was transformed into Agrobacterium strain GV3101. Six 35S::eGFP-CsCBF constructs and the control plasmids without CsCBF coding sequences were separately infiltrated into tobacco (*Nicotiana tabacum*) leaves by the Agrobacterium-mediated genetic transformation method. DAPI (4',6-diamidino-2-phenylindole dihydrochloride) was used to identify nuclei. The cells of transformed tobacco leaves were observed by an Olympus IX81 fluorescence microscope (Olympus, Japan).

### Transactivation Assay Analysis

Each *CsCBF* gene was amplified and cloned into the pGBKT7 vector. Six pGBKT7-CsCBF vectors, the pGBKT7-AtCBF2 vector (positive control) and the pGBKT7 vector (negative control) were separately transformed into the Y2HGold yeast strain. The transformed yeast cells were incubated on SD/-Trp, SD/-Trp/-His-Ade, and SD/-Trp-His-Ade-x-gal plates at 30°C for 3 d.

### RNA Isolation and Quantitative RT-PCR

Total RNA was extracted using the RNAprep Pure Plant Kit (Tiangen, Beijing, China). A Nanodrop 2000 spectrophotometer (Thermo Scientific, Wilmington, USA) was used to measure the concentration of isolated RNA, and the quality was assessed using 1.2% formaldehyde–agarose gel electrophoresis. cDNA was synthesized for qRT-PCR by the PrimeScript™ RT Reagent Kit with gDNA Eraser (Takara, Tokyo, Japan) and diluted 10-fold for PCR amplification.

The specific primers for qRT-PCR were designed by Primer Premier 5 software and synthesized by Sangon Biotech Co. (Shanghai, China). The qRT-PCR reaction program was performed under the following conditions: 95°C for 30 s, followed by 40 cycles at 95°C for 5 s, and 60°C for 30 s. The reaction volume was 25 μL, which contained 4 μL of diluted cDNA, 6.5 μL of deionized water, 12.5 μL of SYBR® Premix Ex Taq™ II (Tli RNaseH Plus; TaKaRa), 1 μL of forward primer, and 1 μL of reverse primer. *CsGAPDH* was used as the reference gene (Pan et al., 2017). The relative gene expression levels were calculated using the comparative 2^−ΔΔCt^ method (Livak and Schmittgen, 2001). Regarding the heatmaps, the 2^−ΔΔCt^ values of the transcripts of the *CsCBF* genes were normalized as the log2-fold change. In the stress-treated plant samples, the values were normalized to plant samples of the 0 h treatment and expressed as a log2-fold change. qRT-PCR experiments were conducted with three independent total RNA samples.

### Functional Analysis of CsCBF3-Overexpressing Transgenic *Arabidopsis thaliana*

Expression of *CsCBF3* shows significant changes during cold treatment and we selected it for overexpression. The ORF of *CsCBF3* was cloned into the pBI121 vector. The construct was transformed into Agrobacterium strain GV3101 by electroporation, and the Arabidopsis plants were transformed using the floral dip method. Arabidopsis ecotype Columbia-0 (col-0) was used as the wild type in this study. Transformed plants were selected on the basis of their resistance to kanamycin, and 4-weeks-old homozygous T3 plants were used for further experiments. Two transgenic lines were treated at −4 and −8°C for 12 h and then grown under 25°C conditions for analysis of survival rate.

## Results

### Identification and Characterization of CBF Genes in Tea Plant

We initially identified 10 *CBF* genes from genome and transcriptome databases of tea plant. Six of them were successfully cloned and deposited into the NCBI GenBank database under the accession numbers *CsCBF1* (EU563238.1), *CsCBF2* (KC702795.1), *CsCBF3* (MH017428.1), *CsCBF4* (KF988866.1), *CsCBF5* (MH165878.1), and *CsCBF6* (MN544638.1). Alignment of the sequences obtained from cDNA and genomic DNA indicated that the *CsCBF* genes were intronless. The CDS length of the *CsCBFs* ranged from 720 to 879 bp, and the genes encoded proteins with lengths varying from 239 to 292 amino acids. The molecular weights were between 26.43 and 32.86 kDa, and the *pI*-values ranged from 4.84 to 8.09. Most of the CsCBF proteins presented grand average hydropathicity (GRAVY) values of <0, implying their hydrophilic nature (Table 1).

**Table 1:** The characteristics of *CsCBFs*.

**Gene symbol**	**Gene identifier^**a**^**	**CDS (bp)**	**Protein (aa)**	**MW (kDa)^**b**^**	**pI^**c**^**	**GRAVY^**d**^**
*CsCBF1*	CSS023229.1	777	259	28.26	5.08	−0.48
*CsCBF2*	CSS018717.1	717	239	26.43	5.22	−0.28
*CsCBF3*	CSS002244.1	774	258	27.78	4.94	−0.47
*CsCBF4*	CSS022600.1	750	250	27.70	5.91	−0.6
*CsCBF5*	CSS001387.1	741	247	27.15	4.87	−0.44
*CsCBF6*	CSS038056.1	900	300	33.63	7.08	−0.52

a *Gene IDs are from C. sinensis var. sinensis genome (marked with CSS) (Xia E. -H. et al., 2019)*.

b *Molecular weight*.

c *Isoelectric point*.

d *Grand average of hydropathicity*.

### Sequence Alignment and Phylogenetic Analysis

**Figure 1:**
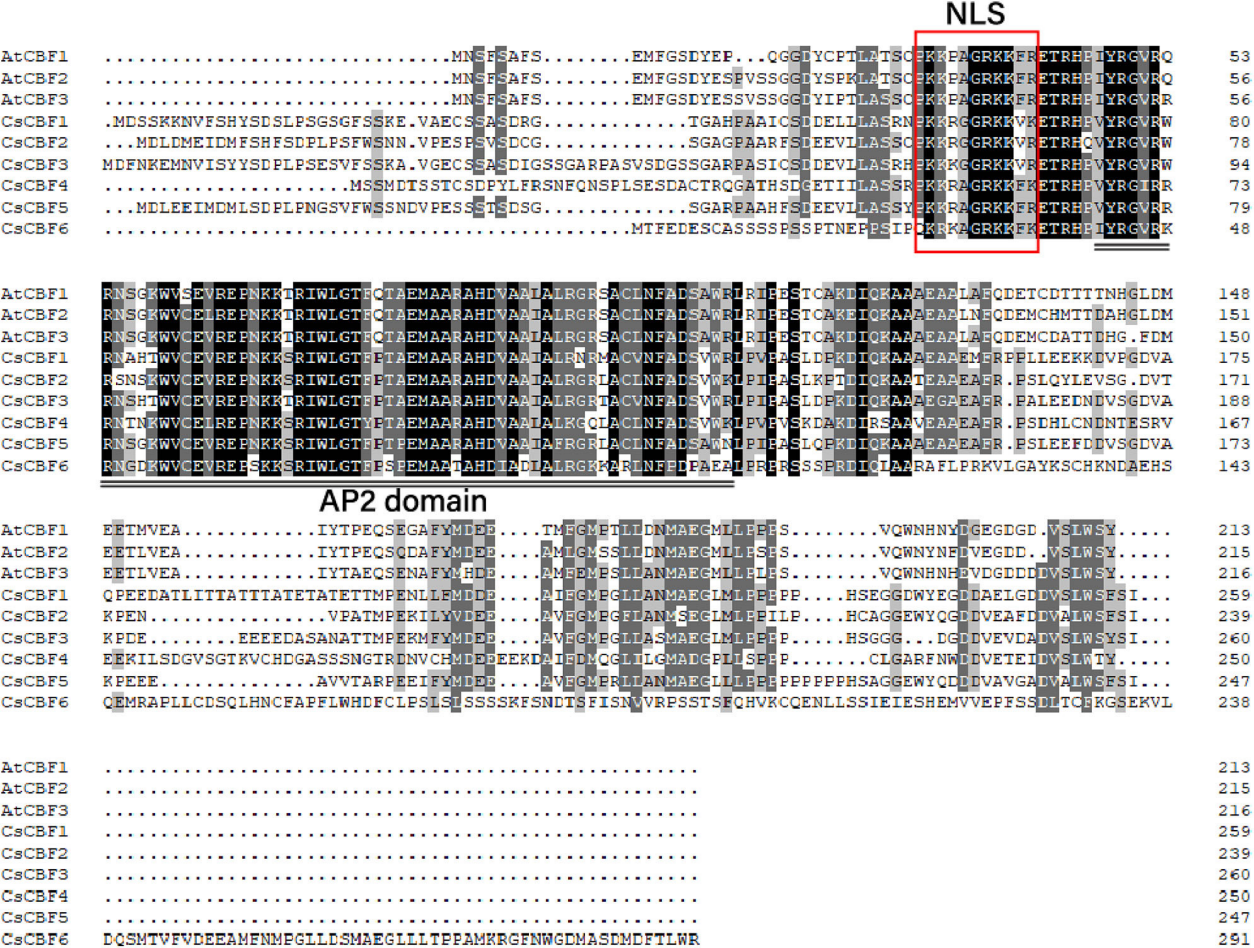
Sequence alignment of the amino acid sequence of CBFs in *C. sinensis* and *Arabidopsis*. Identical and conserved amino acids are shaded in black and gray, respectively. The conserved nuclear localization signal (NLS) and AP2 DNA-binding domain are shown. The NLS sequence is indicated by a red rectangle, and the conserved AP2 domain is underlined (double line).

We investigated the amino acid characteristics of CBF proteins of *C. sinensis* and *A. thaliana*. Alignment of the amino acid sequences of the CsCBFs and AtCBFs revealed that the CsCBFs contained one highly conserved DNA-binding domain (AP2 domain) consisting of 59 amino acids. A putative nuclear localization signal (NLS) sequence (PKKRAGRKKFK) was detected in the N-terminal region. In addition, the C-terminal regions of the CsCBF proteins were highly diverged, particularly CsCBF6 (Figure 1).

**Figure 2:**
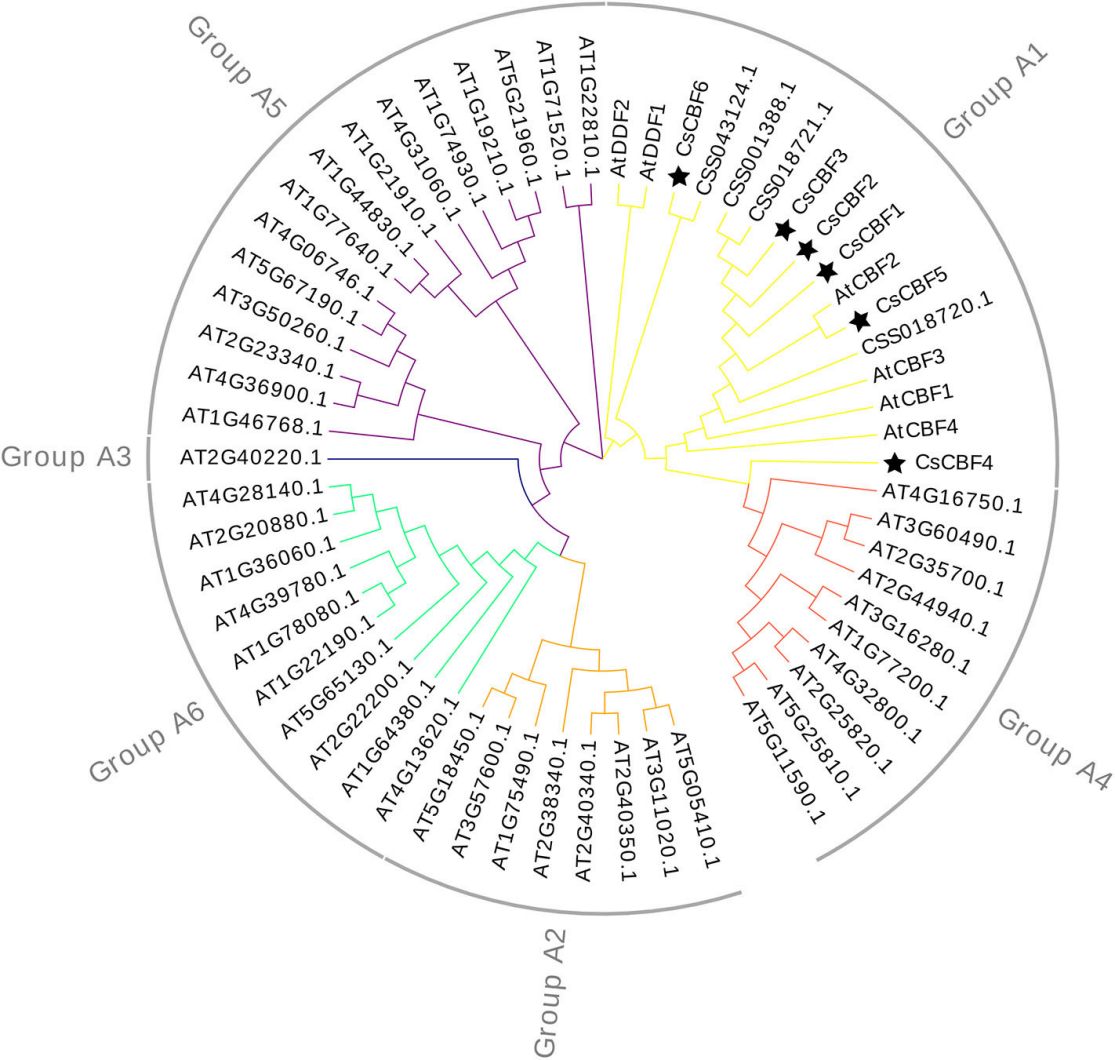
Phylogenetic analysis of CBF proteins from *C. sinen*sis and *Arabidopsis*. All protein sequences are listed in Table S1.

A phylogenetic tree was constructed using the neighbor-joining method with the MEGA program to explore the evolutionary relationships among CBF homologs in *C. sinensis* and *A. thaliana* (Figure 2). The 60 collected DREBs from *C. sinensis* and *A. thaliana* were clustered into six groups. All 10 CsCBF proteins were clustered together with AtDDF1-2 and AtCBF1-4 in Group A1. The result showed that CsCBFs belongs to DREB-A1 subfamily.

### Motif Analysis

**Figure 3:**
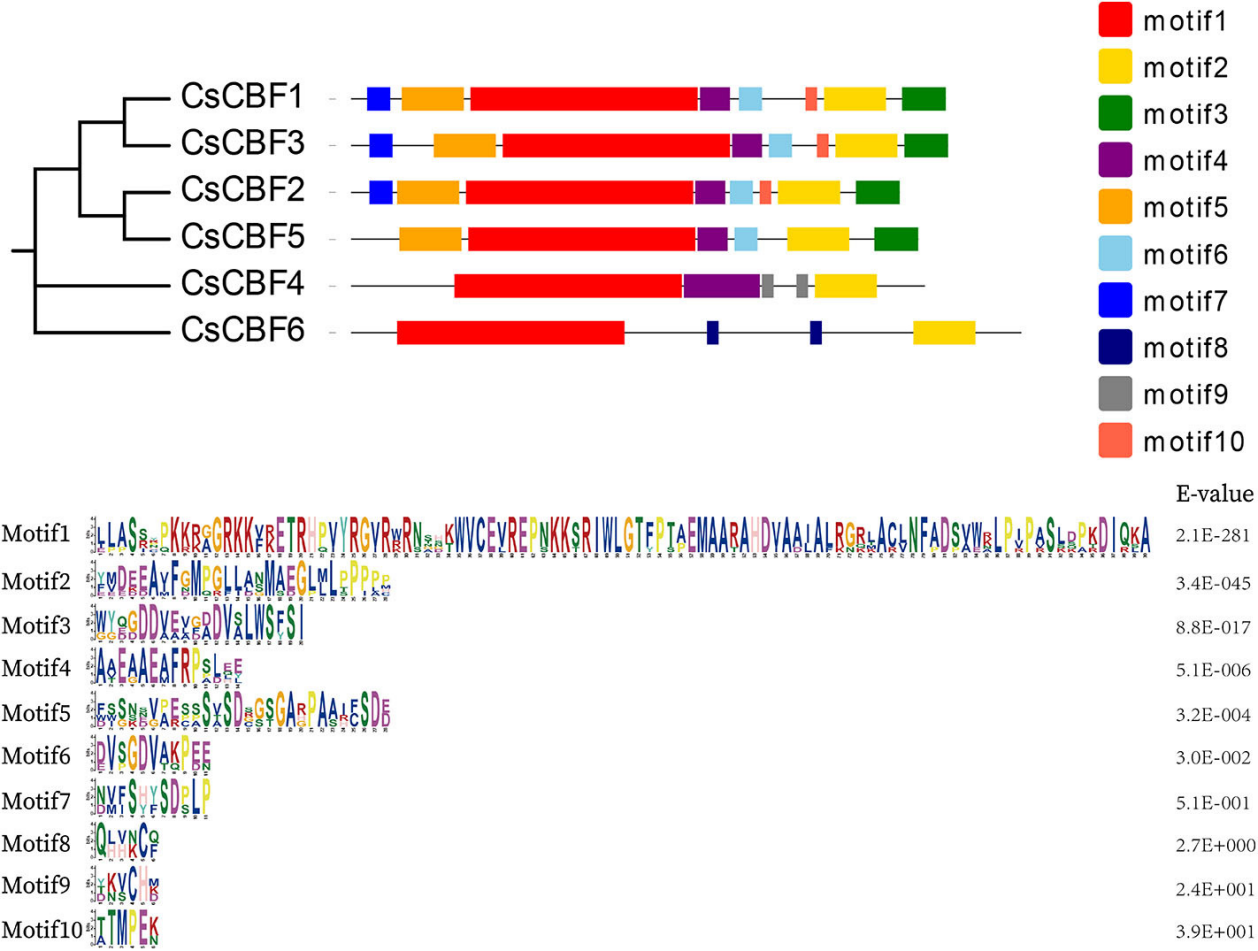
Motif analysis of CsCBF proteins. Different motifs are represented by different colored boxes.

To explore the diversification of *CBF* genes in tea plants, we examined the conserved motifs within the CsCBFs proteins using the MEME program (Figure 3). Ten motifs were identified. Multilevel consensus sequences and the *E*-value of each motif are shown. The results revealed conserved motif distribution among CsCBF proteins. Motif 1 and motif 2 were found in all CBFs. Motifs 3–6 could be found in CsCBF1, 2, 3, and 5. Motif 7 was mainly identified in the C-terminal regions of CBF1, 2, and 3. Motif 8 was unique to CsCBF6 and motif 9 was unique to CsCBF4. Motif 10 was distributed in CBF1, 2, and 3. Motif analysis supports the results of the phylogenetic analysis.

### Subcellular Localization of CsCBF Proteins

**Figure 4:**
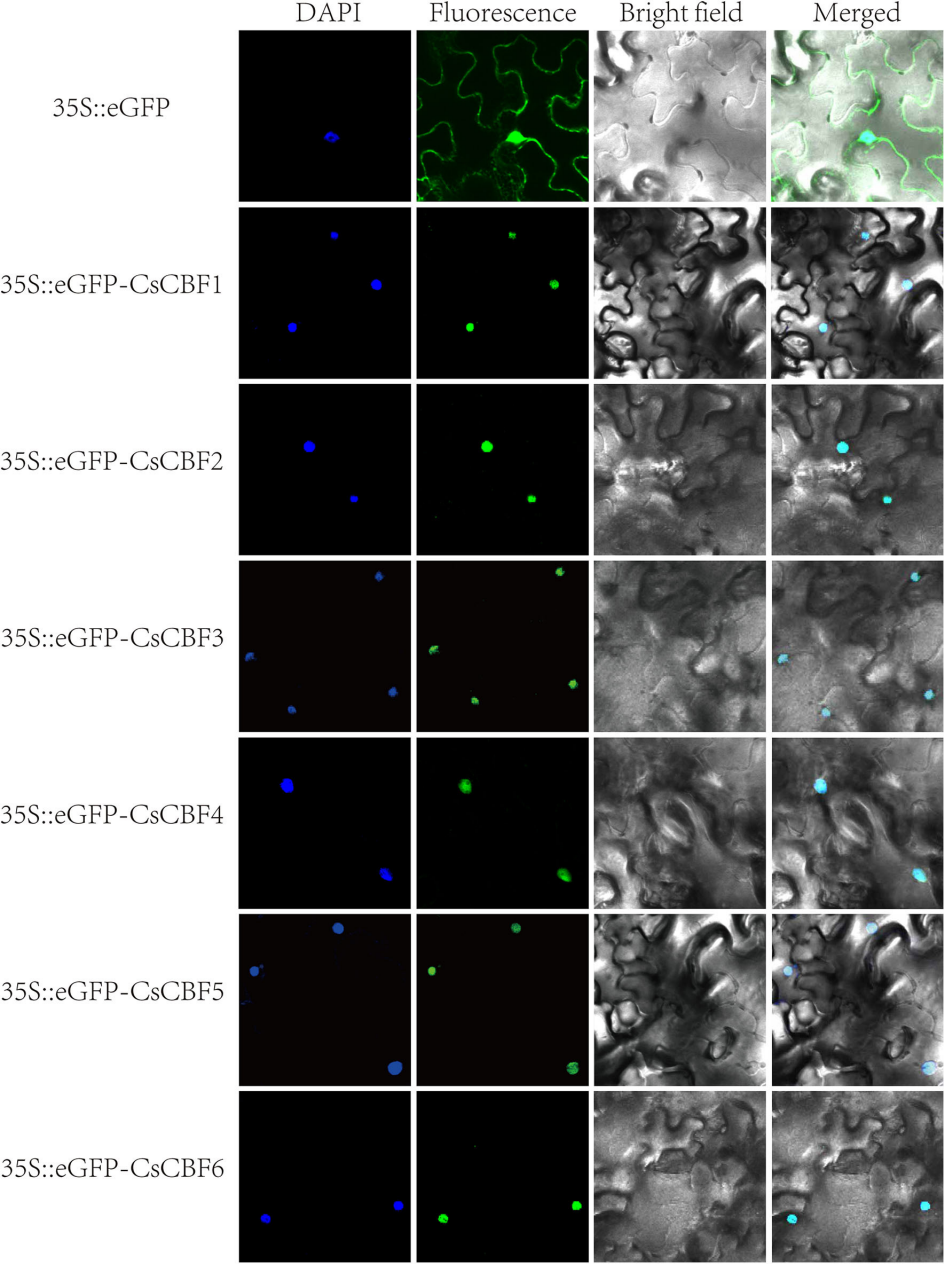
Subcellular localization of CsCBF proteins in tobacco epidermal cells. The 35S::GFP vector was used as a positive control. Images show 4',6-diamino-phenylindole (DAPI) staining fluorescence, GFP fluorescence, and bright light individually and in combination to demonstrate the morphology of the cells.

To investigate the subcellular localization of CsCBF proteins, six 35S::eGFP-CsCBF constructs, one for each CsCBF, and a 35S::eGFP (positive control) construct were generated and transiently expressed in tobacco leaves. As shown in Figure 4, eGFP alone resulted in a diffuse distribution of green fluorescence throughout the entire cell. In contrast, CsCBF-eGFP proteins localized predominantly to the nucleus, which was further confirmed by 4′,6-diamidino-2-phenylindole (DAPI) staining. The results indicated that CsCBFs are nuclear localized proteins.

### Transactivation Assay of CsCBFs

**Figure 5:**
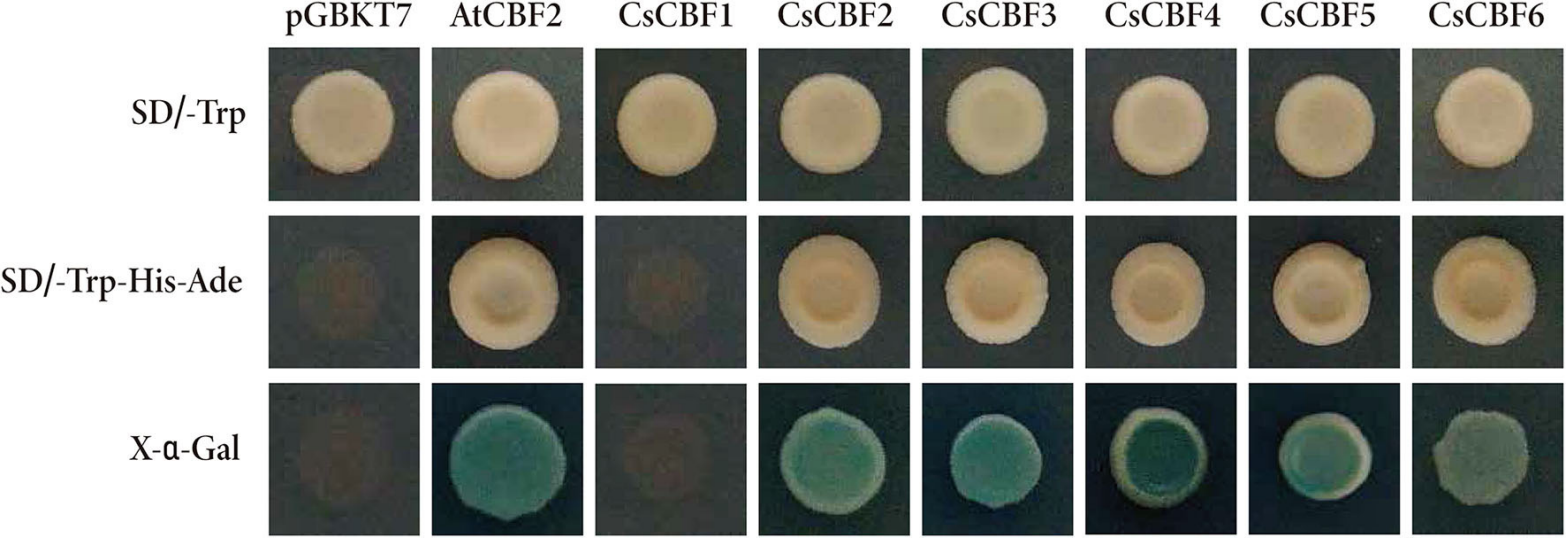
Transcriptional activity analysis of CsCBFs. The entire ORFs of CsCBF sequences were fused to the vector pGBKT7 containing the GAL4 DNA-binding domain. The images in the top row and the middle row show the transformed yeast cells growing on *SD* medium without Trp and *SD* medium without Trp, His, or Ade. The bottom row exhibits the α-galactosidase activity of the transformed yeast cells.

To examine the transcriptional activities of CsCBFs, full length sequences of six CsCBFs and AtCBF2 (positive control) were fused to the vector pGBKT7 containing the GAL4 DNA-binding domain and subsequently transformed into yeast. The yeast cells harboring pGBKT7-CsCBF2, pGBKT7-CsCBF3, pGBKT7-CsCBF4, pGBKT7-CsCBF5, pGBKT7-CsCBF6, and pGBKT7-AtCBF2 grew well on the selection media without Trp, His or adenine (SD/-Trp-His-Ade) and were positive for α-galactosidase activity. The yeast cells with the empty vector pGBKT7 (negative control) and pGBKT7-CsCBF1, which is missing the transcriptional activation domain GAL4 AD, were unable to grow on the same medium. The results indicated that CsCBF2, CsCBF3, CsCBF4, CsCBF5, and CsCBF6 have transcriptional activity, while CsCBF1 has no transcriptional activity (Figure 5).

### *Cis*-Element Analysis of the Promoter Regions of *CsCBF* Genes

To further investigate the regulatory mechanism of *CsCBFs*, sequences 2,000 bp upstream of the start codon of the *CsCBF* genes were extracted from the tea plant genome database (Xia E. et al., 2019). As shown in Table 2, all CsCBF proteins contained the MeJA-responsive element (CGTCA), stress responsiveness element (MYB), and water stress and dehydration responsiveness element (MYC). The abscisic acid-responsive element (ABRE) was found in CsCBF1-pro, CsCBF3-pro, CsCBF4-pro, CsCBF5-pro, and CsCBF6-pro. Ethylene-responsive elements (EREs) were distributed in CsCBF1-pro, CsCBF2-pro, CsCBF3-pro, and CsCBF5-pro. The gibberellin-responsive element (GARE) was unique to CsCBF4-pro. The light-responsive element (G-box) was found in CsCBF1-pro, CsCBF3-pro, CsCBF4-pro, CsCBF5-pro, and CsCBF6-pro. The low-temperature-responsive element (LTR) was shown in CsCBF2-pro, CsCBF4-pro, CsCBF5-pro, and CsCBF6-pro. The Myb-binding site (MBS) was distributed in CsCBF2-pro, CsCBF3-pro, and CsCBF6-pro. Salicylic acid-responsive elements (TCA-element) were unique to CsCBF4-pro and CsCBF5-pro. Defense and stress-responsive elements (TC-rich repeat) were found in CsCBF1-pro, CsCBF5-pro, and CsCBF6-pro. Auxin-responsive elements (TGA elements) were found in CsCBF2-pro and CsCBF4-pro (Table 2).

**Table 2:** The cis-element analysis of *CsCBFs* promoter regions.

**cis-element**	**Putative function**	**CsCBF1**	**CsCBF2**	**CsCBF3**	**CsCBF4**	**CsCBF5**	**CsCBF6**
ABRE	Abscisic acid responsiveness element	1	0	7	15	2	5
CGTCA	MeJA-responsiveness	3	1	3	9	3	1
ERE	Ethylene-responsive element	1	1	6	0	4	0
GARE	Gibberellin-responsive element	0	0	0	1	0	0
G-box	Light responsiveness	1	0	8	18	3	5
LTR	Low-temperature responsiveness	0	1	0	1	2	1
MBS	Myb-binding site involved in drought inducibility	0	1	1	0	0	2
MYB	Stress responsiveness	3	3	2	2	1	2
MYC	Water stress and dehydration responsiveness	3	3	4	3	3	2
TCA-element	Salicylic acid responsiveness	0	0	0	1	1	0
TC-rich repeats	Defense and stress responsiveness	2	0	0	0	1	1
TGA-element	Auxin-responsive element	0	1	0	2	0	0

### Expression Analysis of *CsCBFs* Under Various Abiotic Stresses

**Figure 6:**
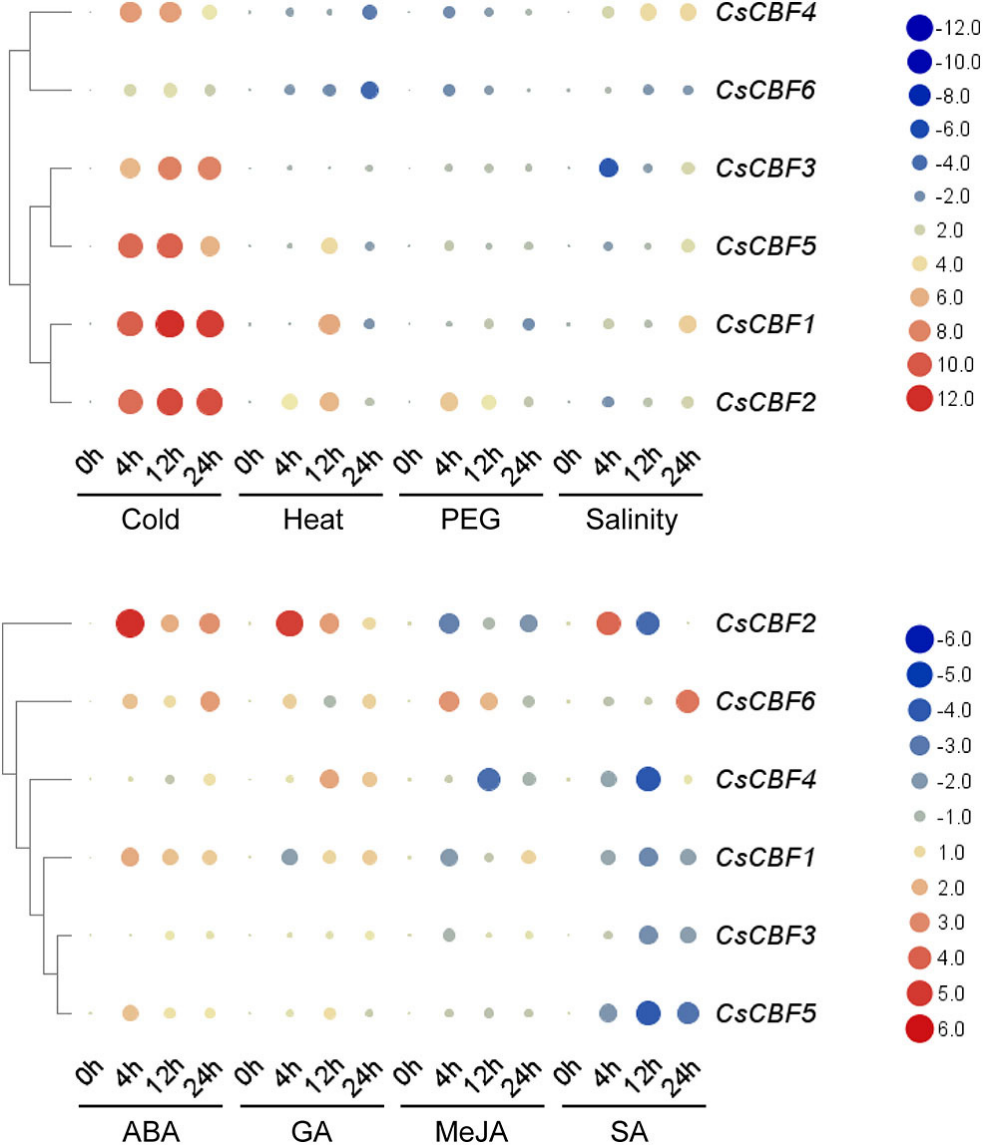
Expression patterns of *CsCBF* genes in response to cold, heat, PEG, salinity, ABA, GA, MeJA, and SA stresses in tea plant cultivars. The relative gene expression levels were calculated using the 2^−ΔΔCt^ method and expressed as the fold change relative to expression of the 0-h treatment. *GAPDH* was used as a housekeeping gene. The mean expression values were again normalized using logarithm with the base of two. The color bar in all heat maps represents the expression values: red represents upregulation, black represents no significant difference in expression, and green denotes downregulation.

We investigated the expression profile of *CsCBF* genes in response to eight stress treatments (cold, heat, PEG, salinity, ABA, GA, MeJA, and SA) by qRT-PCR (Figure 6). Under cold treatment, all *CsCBFs* showed high expression levels during 24 h. Notably, the expression of *CsCBF1,2,3,4*, and *5* was much higher than that of *CsCBF6*. The expression of *CsCBF1, 2*, and *3* increased a 100-fold or even 1000-fold throughout the entire incubation time, while the expression of *CsCBF4, 5*, and *6* increased with processing time but reached a maximum at 12 h. Under heat stress, *CsCBF1, 2*, and *5* presented an expression trend of rising first and then falling, while the expression of *CsCBF4* and *6* declined during processing, and the expression of *CsCBF3* fell to 12 h and then returned to normal. Under PEG stress, *CsCBF2* had a strong increasing response. *CsCBF3* and *5* showed no response to the treatment. The expression of *CsCBF4* and *6* was downregulated and reached a minimum at 12 h, and then the expression level returned to the control level. *CsCBF1* showed a decrease over time. Under salt stress, *CsCBF1* and *4* showed a trend of increasing expression over time. The expression of *CsCBF2, 3*, and *5* first dropped and reached the lowest level at 4 h, then began to rise, and finally was overall positively expressed. *CsCBF6* expression decreased. The results of the treatment of four plant hormones were as follows: under ABA treatment, the expression of *CsCBF1* and *2* increased and peaked at 4 h. *CsCBF3* and *5* had almost no response. *CsCBF4* was downregulated and reached its minimum at 12 h, then it returned to the control level. *CsCBF6* slowly increased with the treatment process. Under GA treatment, *CsCBF1* and *6* both decreased and reached a minimum at 4 and 12 h, respectively, and finally returned to the control level. CsCBF*2* and *4* both increased and reached a maximum at 4 and 12 h, respectively. *CsCBF3* showed no response to GA. *CsCBF5* showed a downward adjustment in the later stages of processing. Under MeJA treatment, the expression of *CsCBF1, 2, 3, 4*, and *5* was downregulated. *CsCBF6* rose in 4 h and then decreased with time. Under SA treatment, *CsCBF1, 3*, and *5* were downregulated and reached a minimum at 12 h. *CsCBF2* was positive in the first 4 h and then decreased to negative over time. *CsCBF4* was downregulated and reached its minimum at 12 h, then it returned to the control level. *CsCBF6* was negatively regulated and then positively regulated.

In general, the results illustrated that cold, ABA and GA could induce high expression of most of the *CsCBF* genes.

### Overexpression of *CsCBF3* in *Arabidopsis thaliana* Improves Tolerance to Cold Stress

**Figure 7:**
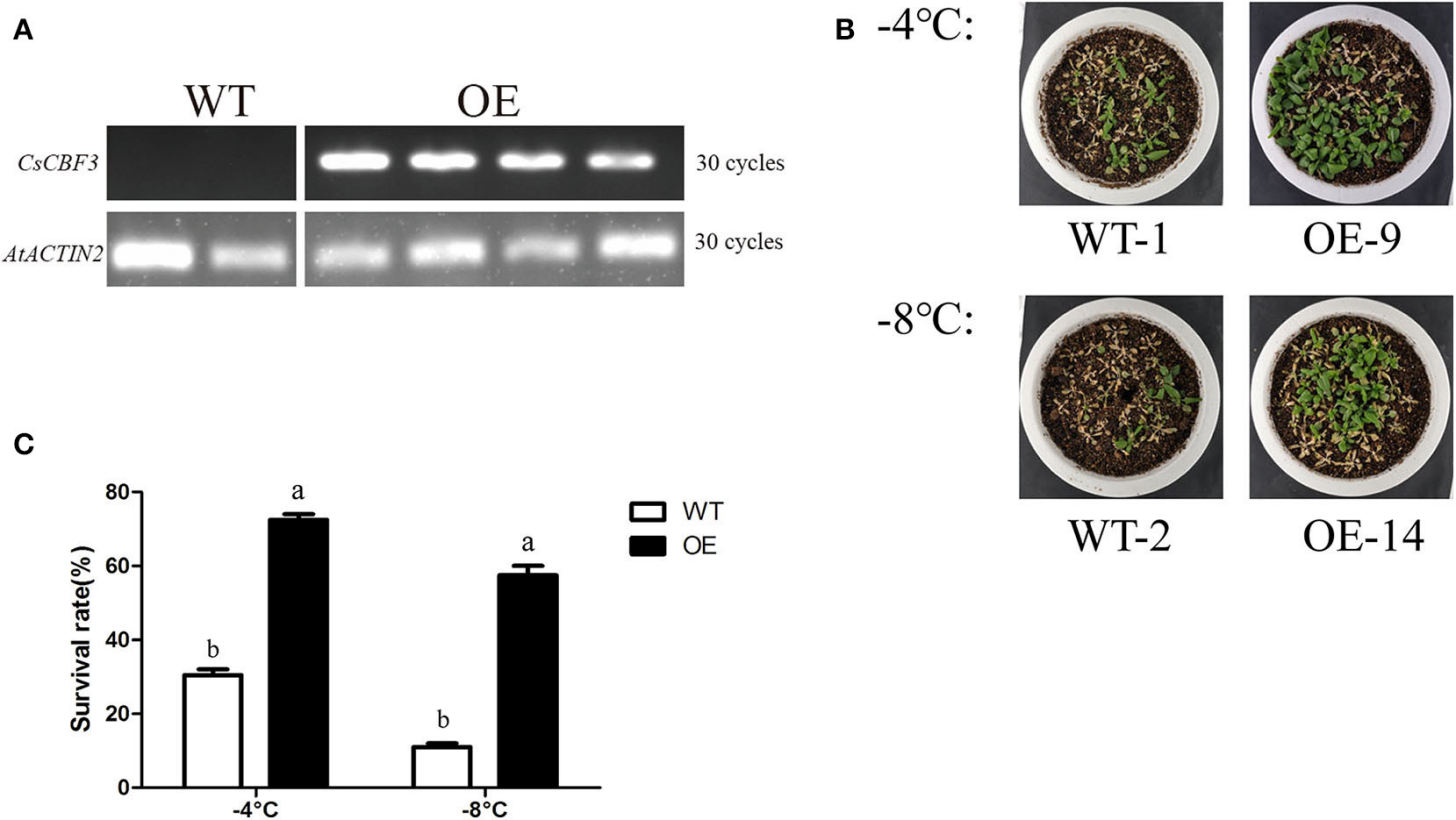
Low-temperature tolerance of transgenic and wild-type *A. thaliana*. **(A)** Expression level of *CsCBF3* in wild-type and transgenic plants. **(B)** A 3-days recovery growing situation of WT and OE plants after −4 and −8°C low temperature treatment. **(C)** Survival rates of wild-type and transgenic plants (*P* < 0.05).

To confirm the *in vivo* functions of the *CsCBF3* gene during low-temperature stress in plants, we transferred *CsCBF3* into *A. thaliana*. The expression of *CsCBF3* was detected using real-time PCR assay in overexpressed (OE) plants but not in wild-type (WT) plants (Figure 7A). Two overexpressed lines (OE-9 and OE-14) were treated in low temperature conditions. Thirty seeds of wild-type and overexpressed plants were selected for treatment. Under normal growth conditions (25°C), there were no obvious differences in survival rate between the WT and transgenic plants. A 3-days recovery after low-temperature treatment at −4 and −8°C for 12 h, the survival rates of WT plants significantly decreased compared to those of transgenic plants (Figure 7B). Recovery for 3 d after −4°C treatment, ~31% of wild-type plants survived, while 73% of OE-9 survived. Recovery for 3 d after −8°C treatment, the survival rates of wild-type plants and OE-14 were 11 and 58%, respectively (Figure 7C).

**Figure 8:**
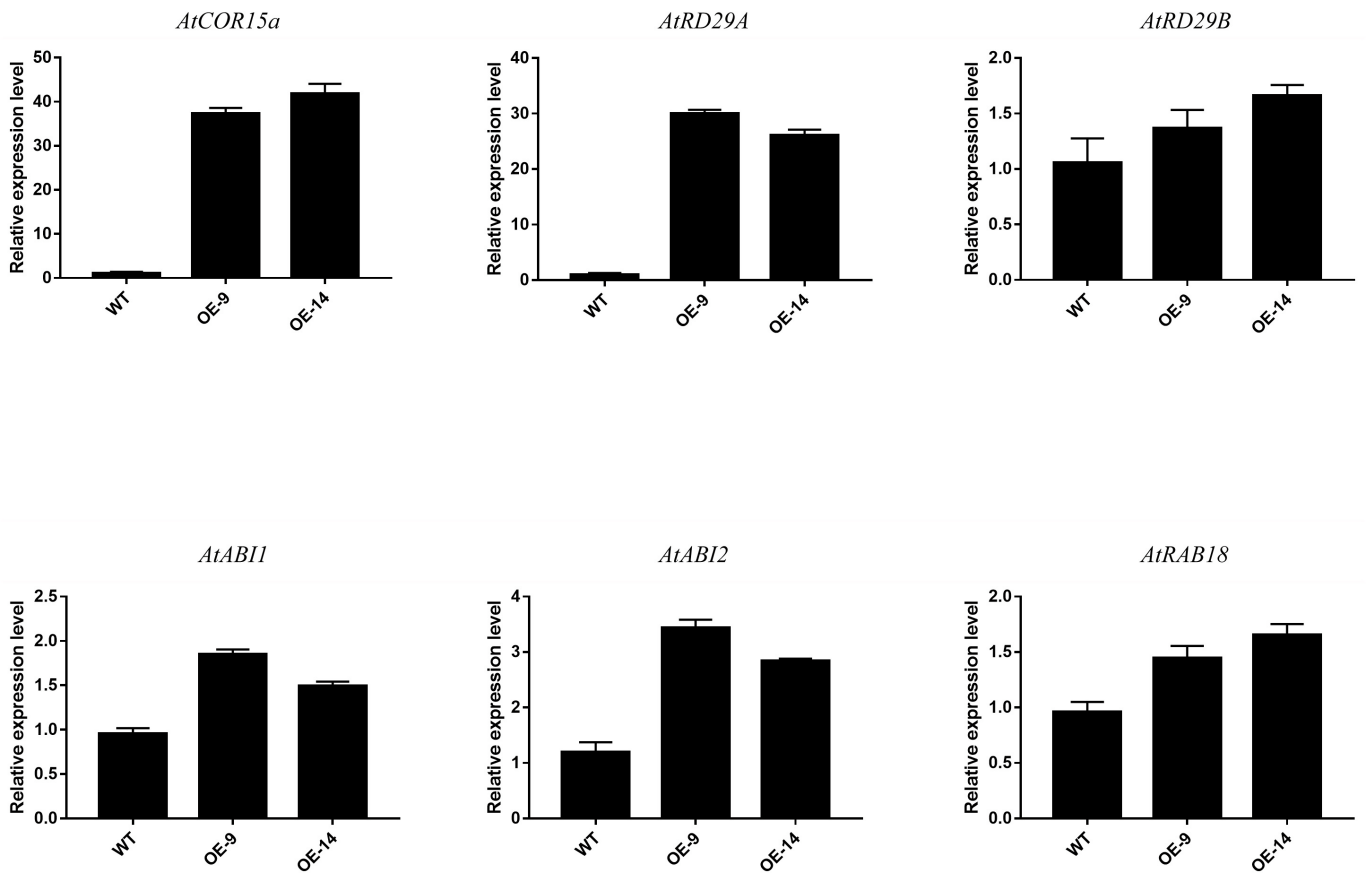
Relative expression level of the downstream target gene of *CsCBF3* in wild-type and transgenic *A. thaliana* plants under cold stress.

In addition, we detected the expression of downstream target ABA-dependent stress-induced genes (*AtRD29B, AtRAB18, AtABI1*, and *AtABI2*) and ABA-independent stress-induced genes (*AtCOR15a* and *AtRD29A*) to explore the potential *CsCBF3*-associated regulatory pathway. Under unstressed conditions, the expression of *AtCOR15a* and *AtRD29A* in transgenic plants was significantly higher than that in wild-type plants. The expression of *AtRD29B, AtRAB18, AtABI1*, and *AtABI2* showed slight differences in WT and OE plants (Figure 8). These results indicated that *CsCBF3* may affect the expression of ABA-independent stress-induced genes to increase plant tolerance to cold stress.

## Discussion

Low temperature is a major abiotic factor that limits crop productivity. When plants suffer from cold stress, CBF/DREB transcription factors are triggered and regulate ~12% of the cold responsive transcriptome, showing important roles in cold tolerance (Sun et al., 2014). To date, two *CsCBF* genes' biological function have been reported in tea plant. Wang et al. (2012) and Ban et al. (2017) found that *CsCBF1* was not expressed at normal temperature (20°C) but was significantly induced at low temperature (4°C). In addition, Wang et al. (2012) used a DNA-binding assay to demonstrate that *CsCBF1* can specifically bind to the CRT/DRE cis-element, suggesting that *CsCBF1* can regulate downstream genes containing CRT elements such as *COR15* and *RD29A*. Wang et al. (2017) showed that overexpression of a *CsDREB* gene in *Arabidopsis thaliana* plants could increase the salt and drought tolerance of transgenic plants. Wang et al. (2019) identified five *CsCBFs* from tea plant genome sequences (Wei et al., 2018), namely *CsCBF1-5*. However, we failed to clone *CsCBF2* (TEA010423), which might be caused by draft nature of the current genome assembly with low assembly quality. The *CsCBF1* identified by Wang et al. (2012) was consistent with that identified in this study but totally different from that identified by Wang et al. (2019) (Data Sheet S3). Similarly, we were not only unable to find homologous gene of the *CsCBF3* identified by Yin et al. (2016) in the current tea plant genome assembly, but also failed to clone it in this study. We here successfully cloned a total of 6 *CsCBFs* (*CsCBF1-6*) based on the latest version of tea plant genome annotation (Xia E. et al., 2019). Of them, *CsCBF1*-*4* were in agreement with those identified by Wang et al. (2019), and *CsCBF5* and *6* were newly discovered (Data Sheet S3). Sequence analysis showed that *CsCBFs* contain an AP2 DNA-binding domain and two signature motifs of the CBF family (Figure 1). These results showed that CsCBF1~6 typically belong to the CBF family. The C-terminus of *CsCBF6* is longer than those of the other *CsCBFs*, and it may cause functional divergence in plants under cold temperatures. Expression of *CsCBF6* is much less than other *CsCBFs* verified this (Figure 6).

It is widely accepted that transcription factors must be present in the nucleus to perform their functions (Wang et al., 2008; Yang et al., 2011). Bioinformatics analysis showed the presence of nuclear localization signal (NLS) sequences in CsCBFs. In our *vivo* targeting experiment using a CsCBFs-fused GFP as a florescent marker demonstrated that the fusion protein was localized to the nucleus of tobacco leaf, suggesting that CsCBFs are nuclear proteins and functions as transcription factors. The results are consistent with the findings in cotton (Shan et al., 2007) and eggplant (Zhou et al., 2018). We designed a yeast single hybrid experiment to verify the transcriptional activities of CsCBFs. The results showed that CsCBF2-6 had transcriptional activity in yeast Y2HGold cells, but not *CsCBF1*. This finding is similar to the findings of Sakuma and Zhao (Sakuma et al., 2006; Zhao X. et al., 2012). A plausible explanation is probably because the secondary structure of the CsCBF1 protein itself is abnormal or CsCBF1 is required to be activated by a posttranslational modification.

CBF encodes a member of the DREB subfamily A-1 of ERF/AP2 transcription factor family. *CBF/DREB* genes from different plant species may have inconsistent expression profiles in response to various stresses (Zhou et al., 2016). There are six members in this subfamily, including *CBF1, CBF2, CBF3*, and *CBF4* in *Arabidopsis thaliana*. *AtCBF1-3* gene is involved in response to low temperature and abscisic acid. *AtCBF4* gene is involved in response to drought stress and abscisic acid treatment, but not to low temperature (Novillo et al., 2004). *EgCBF3* and *FeDREB1* can be upregulated not only by cold but also by osmotic and high-salt stresses (Ebrahimi et al., 2015; Fang et al., 2015). *CaDREBLP1* is not upregulated by low temperature but by dehydration and salt (Hong and Kim, 2005). We examined the expression patterns of *CsCBF* genes in relation to various environmental stresses. At normal growth temperatures, *CsCBF* genes is not transcribed, or is transcribed at lower levels, while *CsCBF* genes except *CsCBF4,6* are rapidly, transiently and strongly induced by cold stress. *CsCBF* genes were induced to varying degrees by other abiotic stress treatment including exposure to high temperature, drought, exogenous hormones, or salinity. *CsCBF4* had a strong response to salinity stress, which was similar to the study by Wang et al. (2017) in *CsDREB*. *AtCBF2* gene is involved in a negative regulatory or feedback circuit of the CBF pathway (CBF2/DREB1C is a negative regulator of CBF1/DREB1B and CBF3/DREB1A expression and plays a central role in stress tolerance in Arabidopsis). Whether there is redundancy or feedback circuit function among *CsCBFs* genes needed to be further research.

Numerous studies have demonstrated that the expression of *CBFs* is regulated by GA, JA, ABA, ETH, and brassinosteroids (BRs) (Shan et al., 2007; Hu et al., 2013; Eremina et al., 2015; Barrero-Gil and Salinas, 2017). Different numbers plant hormone-responsive cis-elements were detected in the *CsCBF* promoters including abscisic acid, MeJA, ethylene, gibberellin, salicylic acid, auxin-responsive element. Results consistent with Wang et al. (2019). The molecular regulatory mechanisms of *CsCBFs* at the crossroads of plant hormone signaling in cold stress response need to be further elucidation. Light responsiveness motifs were also found in *CsCBF* promoters. Previous studies have showed that light is required for many cold-responsive genes, and there is a complex cross-talk between light and cold (Catalá et al., 2011). Large numbers of stress responsiveness cis-elements were found in *CsCBF* promoters, giving the reason tea plant can defend the cold stress. Sequence analysis of the *CsCBF* promoters revealed the existence of different numbers of MYB and MYC binding site, suggesting that induction of the *CsCBF* genes in response to low temperature is involved in the regulation of transcription factors, such as ICE1 and MYB15 in Arabidopsis (Chinnusamy et al., 2003; Agarwal M. et al., 2006).

To confirm *in vivo* functions of the *CsCBF3* gene during low-temperature stress in plants, we ectopically expressed *CsCBF3* into Arabidopsis. The results showed that in the case of *CsCBF3* overexpression, transgenic plants showed enhanced resistance to cold damage. We also observed overexpression of *CsCBF3* resulting in delayed flowering and dwarfism. Under a cold environment, 4-weeks-old overexpression plants had a much higher survival rate than wild-type plants. Similar function was observed in *AtCBF1-3, GhDREB1, SmCBF, LpCBF3* (Xiong and Fei, 2006; Novillo et al., 2007; Shan et al., 2007; Zhou et al., 2018). To clarify how *CsCBF3*-overexpressing transgenic plants cope with low temperature stress, we examined the relative expression level of the downstream target gene of *CBF* identified in Arabidopsis plants. The results showed that the genes belonging to the ABA-independent pathway had a higher expression than the ABA-dependent genes when transgenic Arabidopsis plants were exposed to cold stress. Zhang et al. (2004) demonstrated the *LeCBF1* in a heterologous system could activate the Arabidopsis cold related (COR) genes involved in increasing freezing tolerance, but that *LeCBF1* in tomato plants did not up-regulate equivalent genes (Zhang et al., 2004). The function of *CsCBF3* overexpression in tea plant can't be studied because of genetic transformation system has not been established. We speculated that *CsCBF3* overexpression in transgenic plants improved cold tolerance mainly through the ABA-independent pathway, which was consistent with CBFs belonging to the ABA-independent pathway.

## Conclusion

CBF/DREB transcription factors were identified in tea plants. CsCBF proteins were localized to the nucleus. CsCBFs had transcriptional activity except CsCBF1. *CsCBF* gene expression could be affected by abiotic stress and plant hormones. Ectopic expression of *CsCBF3* in Arabidopsis induced cold tolerance, and the mechanism of *CsCBF3* regulation of downstream target genes was mainly the ABA-independent pathway.

## Data Availability Statement

The datasets generated for this study can be found in the NCBI, CsCBF1 (EU563238.1), CsCBF2 (KC702795.1), CsCBF3 (MH017428.1), CsCBF4 (KF988866.1), CsCBF5 (MH165878.1), CsCBF6 (MN544638.1).

## Author Contributions

YL and EX designed the study. ZH, QB, JH, XZ, YC, JM, and ML conducted the experiments and analyzed the data. ZH and QB prepared the manuscript. All authors consent to the manuscript.

## Conflict of Interest

The authors declare that the research was conducted in the absence of any commercial or financial relationships that could be construed as a potential conflict of interest.

## Supplementary Material

Table S1 Primers used in this study.

Table S2 Name of genes in this study.

Data Sheet S1 The amino acid sequences of CBF genes used in phylogenetic tree construction.

Data Sheet S2 Expression level of *CsCBFs* under abiotic stresses.

Data Sheet S3 Expression level of downstream target genes of CsCBF3 in Arabidopsis.
