# Supplementary material for: Genome-Wide Characterization of the C-repeat Binding Factor (CBF) Gene Family Involved in the Response to Abiotic Stresses in Tea Plant (Camellia sinensis)
Source: Front Plant Sci. 2020 Jul 23;11:921. doi: 10.3389/fpls.2020.00921 (PMC7396485; doi:10.3389/fpls.2020.00921)
Supplement: Table S1 — Primers used in this study. [file Table_1.docx]

Table S1

Primers used in this study

| primer | Primer sequence (5’–3’) |
| --- | --- |
| CsCBF1-F | ATGGATTCGAGCAAGAAAAATGT |
| CsCBF1-R | TTAAATTGAGAAGCTCCAGAGTGACA |
| CsCBF2-F | ATGGATTTGGACATGGAAATCG |
| CsCBF2-R | TTAAATTGAGAAGCTCCAGAGTGC |
| CsCBF3-F | ATGGATTTCAACAAAGAAATGAATGT |
| CsCBF3-R | TTAAATTGAGTAGCTCCAGAGTGACAC |
| CsCBF4-F | ATGTCATCGATGGATACTTCGAGTAC |
| CsCBF4-R | TTAGTAAGTCCACAATGAAACATCAATC |
| CsCBF5-F | ATGGATTTGGAAGAAATAATGGATATG |
| CsCBF5-R | TTAAATAGAAAAGCTCCAGAGTGCC |
| CsCBF6-F | ATGACTTTTGAAGATGAGTCATGTGC |
| CsCBF6-R | TCAGTATCTCCATAAAGTGAAGTCCAT |
| pK7WGF2.0-CsCBF1-F | GGGGACAAGTTTGTACAAAAAAGCAGGCTTCATGGATTCGAGCAAGAAAAATGT |
| pK7WGF2.0-CsCBF1-R | GGGGACCACTTTGTACAAGAAAGCTGGGTCTTAAATTGAGAAGCTCCAGAGTGACA |
| pK7WGF2.0-CsCBF2-F | GGGGACAAGTTTGTACAAAAAAGCAGGCTTCATGGATTTGGACATGGAAATCG |
| pK7WGF2.0-CsCBF2-R | GGGGACCACTTTGTACAAGAAAGCTGGGTCTTAAATTGAGAAGCTCCAGAGTGC |
| pK7WGF2.0-CsCBF3-F | GGGGACAAGTTTGTACAAAAAAGCAGGCTTCATGGATTTCAACAAAGAAATGAATGT |
| pK7WGF2.0-CsCBF3-R | GGGGACCACTTTGTACAAGAAAGCTGGGTCTTAAATTGAGTAGCTCCAGAGTGACAC |
| pK7WGF2.0-CsCBF4-F | GGGGACAAGTTTGTACAAAAAAGCAGGCTTCATGTCATCGATGGATACTTCGAGTAC |
| pK7WGF2.0-CsCBF4-R | GGGGACCACTTTGTACAAGAAAGCTGGGTCTTAGTAAGTCCACAATGAAACATCAATC |
| pK7WGF2.0-CsCBF5-F | GGGGACAAGTTTGTACAAAAAAGCAGGCTTCATGGATTTGGAAGAAATAATGGATATG |
| pK7WGF2.0-CsCBF5-R | GGGGACCACTTTGTACAAGAAAGCTGGGTCTTAAATAGAAAAGCTCCAGAGTGCC |
| pK7WGF2.0-CsCBF6-F | GGGGACAAGTTTGTACAAAAAAGCAGGCTTCATGACTTTTGAAGATGAGTCATGTGC |
| pK7WGF2.0-CsCBF6-R | GGGGACCACTTTGTACAAGAAAGCTGGGTCTCAGTATCTCCATAAAGTGAAGTCCAT |
| pGBKT7-CsCBF1-F | ATGGCCATGGAGGCCGAATTCATGGATTCGAGCAAGAAAAATGT |
| pGBKT7-CsCBF1-R | CCGCTGCAGGTCGACGGATCCTTAAATTGAGAAGCTCCAGAGTGACA |
| pGBKT7-CsCBF2-F | ATGGCCATGGAGGCCGAATTCATGGATTTGGACATGGAAATCG |
| pGBKT7-CsCBF2-R | CCGCTGCAGGTCGACGGATCCTTAAATTGAGAAGCTCCAGAGTGC |
| pGBKT7-CsCBF3-F | ATGGCCATGGAGGCCGAATTCATGGATTTCAACAAAGAAATGAATGT |
| pGBKT7-CsCBF3-R | CCGCTGCAGGTCGACGGATCCTTAAATTGAGTAGCTCCAGAGTGACAC |
| pGBKT7-CsCBF4-F | ATGGCCATGGAGGCCGAATTCATGTCATCGATGGATACTTCGAGTAC |
| pGBKT7-CsCBF4-R | CCGCTGCAGGTCGACGGATCCTTAGTAAGTCCACAATGAAACATCAATC |
| pGBKT7-CsCBF5-F | ATGGCCATGGAGGCCGAATTCATGGATTTGGAAGAAATAATGGATATG |
| pGBKT7-CsCBF5-R | CCGCTGCAGGTCGACGGATCCTTAAATAGAAAAGCTCCAGAGTGCC |
| pGBKT7-CsCBF6-F | ATGGCCATGGAGGCCGAATTCATGACTTTTGAAGATGAGTCATGTGC |
| pGBKT7-CsCBF6-R | CCGCTGCAGGTCGACGGATCCTCAGTATCTCCATAAAGTGAAGTCCAT |
| pGBKT7-AtCBF2-F | ATGGCCATGGAGGCCGAATTCATGAACTCATTTTCTGCCTTTTCTG |
| pGBKT7-AtCBF2-R | CCGCTGCAGGTCGACGGATCCTTAATAGCTCCATAAGGACACGTCA |
| pBI121-CsCBF3-F | GAGAACACGGGGGACTCTAGAATGGATTTCAACAAAGAAACG |
| pBI121-CsCBF3-R | GGACTGACCACCCGGGGATCCTAAATTGAGTAGCTCCAGAGTG |
| CsActin-qPCR-F | GCCATCTTTGATTGGAATGG |
| CsActin-qPCR-R | GGTGCCACAACCTTGATCTT |
| CsCBF1-qPCR-F | GGAATGCTCATCGGCGTCAGAT |
| CsCBF1-qPCR-R | CCACCTCACACCTCGGTACACT |
| CsCBF2-qPCR-F | AGGTGTCAGGTGGAGGAGCAA |
| CsCBF2-qPCR-R | CAATCGCAGCAACGTCGTGAG |
| CsCBF3-qPCR-F | AGCCAGATGAGGAGGAGGAGGA |
| CsCBF3-qPCR-R | GAGTGTGGTGGAGGAGGTAGCA |
| CsCBF4-qPCR-F | AGGAGGAGGAACACCAACAAGT |
| CsCBF4-qPCR-R | TCGCAGCCATCTCAGCAGTT |
| CsCBF5-qPCR-F | CCGAAGAAGCGAGCAGGTAGGA |
| CsCBF5-qPCR-R | GCGGACTTCACAGACCCACTTG |
| CsCBF6-qPCR-F | ACGCCACCCGATTTACAGAGG |
| CsCBF6-qPCR-R | CGTGAGCCGTAGCAGCCATT |
| AtRD29A-qPCR-F | TATTCGCCGGAATCTGACGG |
| AtRD29A-qPCR-R | GATGCCTCACCGTATCCAGG |
| AtCOR15a-qPCR-F | CCTCAACGAGGCCACAAAGA |
| AtCOR15a-qPCR-R | TTTCTCCTCCACATACGCCG |
| AtRD29B-qPCR-F | TAATGTTCCGGCATCGGAGG |
| AtRD29B-qPCR-R | TTCCCAGTCCGATGTTTCCG |
| AtRAB18-qPCR-F | GGCATAGACTTTGCTCGGGA |
| AtRAB18-qPCR-R | CAGCAGTAAGACTCGGCGTT |
| AtABI1-qPCR-F | CAGTGGAATGGAGCTCGTGT |
| AtABI1-qPCR-R | TTGCCATCTCACACGCTTCT |
| AtABI2-qPCR-F | AACCGGAGTTTTGTGACGGT |
| AtABI2-qPCR-R | CCGCAATTCGCGACAAAGAT |
| AtACTIN2-qPCR-F | CTCAGGTATCGCTGACCGTA |
| AtACTIN2-qPCR-R | GATCCACATCTGCTGGAATG |
