# Supplementary material for: Genome-Wide Characterization of the C-repeat Binding Factor (CBF) Gene Family Involved in the Response to Abiotic Stresses in Tea Plant (Camellia sinensis)
Source: Front Plant Sci. 2020 Jul 23;11:921. doi: 10.3389/fpls.2020.00921 (PMC7396485; doi:10.3389/fpls.2020.00921)
Supplement: Table S2 — Name of genes in this study. [file Table_2.docx]

Table S2

| Gene in this study | Gene ID from Wei et al., 2018 | Gene ID from Xia et al., 2019 | Named by Wang et al., 2019 | Named by Wang et al., 2012 | Named by Yin et al., 2016 |
| --- | --- | --- | --- | --- | --- |
| *CsCBF1* | TEA031249.1 | CSS023229.1 |  | *CsCBF1* |  |
| *CsCBF2* | TEA010420.1 | CSS018717.1 | *CsCBF1* |  |  |
| *CsCBF3* | TEA011105.1 | CSS002244.1 | *CsCBF4* |  |  |
| *CsCBF4* | TEA010806.1 | CSS022600.1 | *CsCBF3* |  |  |
| *CsCBF5* | Scaffold433: 3184726:3185465 | CSS001387.1 | *CsCBF1* |  |  |
| *CsCBF6* | TEA015323.1 | CSS038056.1 |  |  |  |
| Not cloned | TEA010423.1 |  | *CsCBF2* |  |  |
| Not found and cloned |  |  |  |  | *CsCBF3* |

Name of gene in this study
