# Supplementary material for: Genome-Wide Characterization of the C-repeat Binding Factor (CBF) Gene Family Involved in the Response to Abiotic Stresses in Tea Plant (Camellia sinensis)
Source: Front Plant Sci. 2020 Jul 23;11:921. doi: 10.3389/fpls.2020.00921 (PMC7396485; doi:10.3389/fpls.2020.00921)
Supplement: Data Sheet S1 — The amino acid sequences of CBF genes used in phylogenetic tree construction. [file Data_Sheet_1.docx]

>AtCBF2

MNSFSAFSEMFGSDYESPVSSGGDYSPKLATSCPKKPAGRKKFRETRHPIYRGVRQRNSGKWVCELREPNKKTRIWLGTF

QTAEMAARAHDVAAIALRGRSACLNFADSAWRLRIPESTCAKEIQKAAAEAALNFQDEMCHMTTDAHGLDMEETLVEAIY

TPEQSQDAFYMDEEAMLGMSSLLDNMAEGMLLPSPSVQWNYNFDVEGDDDVSLWSY

>AtCBF3

MNSFSAFSEMFGSDYESSVSSGGDYIPTLASSCPKKPAGRKKFRETRHPIYRGVRRRNSGKWVCEVREPNKKTRIWLGTF

QTAEMAARAHDVAALALRGRSACLNFADSAWRLRIPESTCAKDIQKAAAEAALAFQDEMCDATTDHGFDMEETLVEAIYT

AEQSENAFYMHDEAMFEMPSLLANMAEGMLLPLPSVQWNHNHEVDGDDDDVSLWSY

>AtCBF1

MNSFSAFSEMFGSDYEPQGGDYCPTLATSCPKKPAGRKKFRETRHPIYRGVRQRNSGKWVSEVREPNKKTRIWLGTFQTA

EMAARAHDVAALALRGRSACLNFADSAWRLRIPESTCAKDIQKAAAEAALAFQDETCDTTTTNHGLDMEETMVEAIYTPE

QSEGAFYMDEETMFGMPTLLDNMAEGMLLPPPSVQWNHNYDGEGDGDVSLWSY

>AtDDF2

MENDDITVAEMKPKKRAGRRIFKETRHPIYRGVRRRDGDKWVCEVREPIHQRRVWLGTYPTADMAARAHDVAVLALRGRS

ACLNFSDSAWRLPVPASTDPDTIRRTAAEAAEMFRPPEFSTGITVLPSASEFDTSDEGVAGMMMRLAEEPLMSPPRSYID

MNTSVYVDEEMCYEDLSLWSY

>AtDDF1

MNNDDIILAEMRPKKRAGRRVFKETRHPVYRGIRRRNGDKWVCEVREPTHQRRIWLGTYPTADMAARAHDVAVLALRGRS

ACLNFADSAWRLPVPESNDPDVIRRVAAEAAEMFRPVDLESGITVLPCAGDDVDLGFGSGSGSGSGSEERNSSSYGFGDY

EEVSTTMMRLAEGPLMSPPRSYMEDMTPTNVYTEEEMCYEDMSLWSYRY

>AtCBF4

MNPFYSTFPDSFLSISDHRSPVSDSSECSPKLASSCPKKRAGRKKFRETRHPIYRGVRQRNSGKWVCEVREPNKKSRIWL

GTFPTVEMAARAHDVAALALRGRSACLNFADSAWRLRIPETTCPKEIQKAASEAAMAFQNETTTEGSKTAAEAEEAAGEG

VREGERRAEEQNGGVFYMDDEALLGMPNFFENMAEGMLLPPPEVGWNHNDFDGVGDVSLWSFDE

>CsCBF1

MDSSKKNVFSHYSDSLPSGSGFSSKEVAECSSASDRGTGAHPAAICSDDELLLASRNPKKRGGRKKVKETRHPVYRGVRWRNAHTWVCEVREPNKKSRIWLGTFPTAEMAARAHDVAAIALRNRMACVNFADSVWRLPVPASLDPKDIQKAAAEAAEMFRPPLLEEKKDVPGDVAQPEEDATLITTATTTATETATETTMPENLLFMDDEAIFGMPGLLANMAEGLMLPPPPPHSEGGDWYEGDDAELGDDVSLWSFSI.

>CsCBF2

MDLDMEIDMFSHFSDPLPSFWSNNVPESPSVSDCGSGAGPAARFSDEEVLLASSCPKKRGGRKKVRETRHQVYRGVRWRSNSKWVCEVREPNKKSRIWLGTFPTAEMAARAHDVAAIALRGRLACLNFADSVWKLPIPASLKPTDIQKAATEAAEAFRPSLQYLEVSGDVTKPENVPATMPEKILYVDEEAVFGMPGFLANMSEGLMLPPILPHCAGGEWYQGDDVEAFDDVALWSFSI.

>CsCBF5

MDLEEIMDMLSDPLPNGSVFWSSNDVPESSSTSDSGSGARPAAHFSDEEVLLASSYPKKRAGRKKFRETRHPVYRGVRRRNSGKWVCEVREPNKKSRIWLGTFPTPEMAARAHDVAAIAFRGRLACLNFADSAWNLPIPASLQPKDIQKAAAEAAEAFRPSLEEFDDVSGDVAKPEEEAVVTARPEEIFYMDEEAVFGMPRLLANMAEGLLLPPPPPPPPPHSAGGEWYQDDDVAVGADVALWSFSI.

>CsCBF4

MSSMDTSSTCSDPYLFRSNFQNSPLSESDACTRQGATHSDGETIILASSRPKKRAGRKKFKETRHPVYRGIRRRNTNKWVCELREPNKKSRIWLGTYPTAEMAARAHDVAALALKGQLACLNFADSVWKLPVPVSKDAKDIRSAAVEAAEAFRPSDHLCNDNTESRVEEKILSDGVSGTKVCHDGASSSNGTRDNVCHMDEEEEKDAIFDMQGLILGMADGPLLSPPPCLGARFNWDDVETEIDVSLWTY.

>CsCBF3

MDFNKEKNVISYYSDPLPSESVFSSKAVGECSSASDIGSSGARPASICSDDEVLLASRHPKKKGGRKKVRETRHPVYRGVRWRNSHTWVCEVREPNKKTRIWLGTFPTAEMAARAHDVAAIALRGRTACVNFADSVWRLPIPASLDPKDIQKAAAEGAEAFRPALEEDNDVSGDVAKPDEEEEEEEEEEAAANATTMPEKVFYMDEEAVFGMPGLLASMAEGLMLPPPPHSAGGDGDDVEVDADVSLWSYSI.

>CsCBF6

MTFEDESCASSSSPSSPTNEPPSIPQKRKAGRKKFKETRHPIYRGVRKRNGDKWVCEVREPSKKSRIWLGTFPSPEMAAT

AHDIADLALRGKKARLNFPDSAEALPRPRSSSPRDIQLAARAFLPRKVLGAYKSCHKNDAEHSQEMRAPLLCDSQPHNYS

APFLWHDFCVPSLSLLSSSKFSNDTSFISSVVRPSSTSFQHVKCQENLLLEPPLADSSSIEIESHEMVVEPFSSDLTCFK

GSEKVLDQSMTVFVDEEAMFNMPGLLDSMAEGLLLTPPAMKRGFNWGDMASDMDFTLWRY

>CSS001388.1

MLVGQFYHPTTLWGNPHWCSTNCIVSDDEVLFPSKYPKKIGGRKKVRENRHPTYRGVRWR

NSHKIWLSTFPIAEMAARTHNFVAIVLKAWKLSIPASLETNDNQKAAVKEVEAFCPSLEE

CEDVSGDVANIGGNGDDDNARGHVFTWGMRQFSGCPDYLQTWQKG*

>CSS018720.1

MCSHFSDPLPSFGSNDVPESPSVSDCGSGARPAARFSDKEVLLASSCPKKRGGRKKVRET

RHQVYRGVKLRNNSIWVCEVREPIKKSRIWLGTFPTAEMAARAHDVAAIALRGRLACLNF

ADSAWKLPVPASLKPKDIKKAATEAAEAFRPSLPYLEVSGDVTKPENLPATMPGENILH*

>CSS018721.1

MCVSGPTTSRNLSVSDCGSGAWPAARFSDEEVLLASSCPKKKGGRKKVRETRHQVYRGVR

PRNNSKWVCEVREPNKKSRIWLGTFLTAEMAARAHDVAAIALRASYSGLIEAKDIQKAAT

EATEAFRPSLQYLEVSGDVTKPENVQATMPEKILYIDEDAVFGMPGLLAKHGRRIDATST

MDDN*

>CSS043124.1

MAARTHDVAVLALRGKMTKLNFSDSTRVLPGAKLSSARDIQMAILEATRTFQSSSTSSPF

NSSRNTNLRLSSISSTELATCAITGSDKGLDSSIELFLDEEAFFNMPGLLNSMAEGLILT

PPALKRGFNWDDTGCDMDMTLWRD*

>AT3G16280.1

MTSLNSSASPTSSSSDQSDATTTTSTHLSEEEAPPRNNNTRKRRRDSSSASSSSSMQHPVYRGVRMRSWGKWVSEIRQPR

KKTRIWLGTFVTADMAARAHDVAALTIKGSSAVLNFPELASLFPRPASSSPHDIQTAAAEAAAMVVEEKLLEKDEAPEAP

PSSESSYVAAESEDEERLEKIVELPNIEEGSYDESVTSRADLAYSEPFDCWVYPPVMDFYEEISEFNFVELWSFNH

>AT2G44940.1

MARQINIESSVSQVTFISSAIPAVSSSSSITASASLSSSPTTSSSSSSSTNSNFIEEDNSKRKASRRSLSSLVSVEDDDD

QNGGGGKRRKTNGGDKHPTYRGVRMRSWGKWVSEIREPRKKSRIWLGTYPTAEMAARAHDVAALAIKGTTAYLNFPKLAG

ELPRPVTNSPKDIQAAASLAAVNWQDSVNDVSNSEVAEIVEAEPSRAVVAQLFSSDTSTTTTTQSQEYSEASCASTSACT

DKDSEEEKLFDLPDLFTDENEMMIRNDAFCYYSSTWQLCGADAGFRLEEPFFLSE

>AT3G60490.1

MGKQINIESSATHHQDNIVSVITATISSSSVVTSSSDSWSTSKRSLVQDNDSGGKRRKSNVSDDNKNPTSYRGVRMRSWG

KWVSEIREPRKKSRIWLGTYPTAEMAARAHDVAALAIKGNSGFLNFPELSGLLPRPVSCSPKDIQAAATKAAEATTWHKP

VIDKKLADELSHSELLSTAQSSTSSSFVFSSDTSETSSTDKESNEETVFDLPDLFTDGLMNPNDAFCLCNGTFTWQLYGE

EDVGFRFEEPFNWQND

>AT4G32800.1

MADSSSDKEKKENNKQPVYRGVRMRSWGKWVSEIREPRKKSRIWLGTFPTAEMAMRAHDVAAMSIKGTSAILNFPELSKL

LPRPVSLSPRDVRAAATKAALMDFDTTAFRSDTETSETTTSNKMSESSESNETVSFSSSSWSSVTSIEESTVSDDLDEIV

KLPSLGTSLNESNEFVIFDSLEDLVYMPRWLSGTEEEVFTYNNNDSSLNYSSVFESWKHFP

>AT2G25820.1

MVDSHGSDTECSSKKKKEKTKEKGVYRGARMRSWGKWVSEIREPRKKSRIWLGTFPTAEMAARAHDVAALSIKGSSAILN

FPELADFLPRPVSLSQQDIQAAAAEAALMDFKTVPFHLQDDSTPLQTRCDTEKIEKWSSSSSSASSSSSSSSSSSSSMLS

GELGDIVELPSLENNVKYDCALYDSLEGLVSMPPWLDATENDFRYGDDSVLLDPCLKESFLWNYE\

>AT4G16750.1

MQDSSSHESQRNLRSPVPEKTGKSSKTKNEQKGVSKQPNFRGVRMRQWGKWVSEIREPRKKSRIWLGTFSTPEMAARAHD

VAALAIKGGSAHLNFPELAYHLPRPASADPKDIQEAAAAAAAVDWKAPESPSSTVTSSPVADDAFSDLPDLLLDVNDHNK

NDGFWDSFPYEDPFFLENY

>AT2G35700.1

MERDDCRRFQDSPAQTTERRVKYKPKKKRAKDDDDEKVVSKHPNFRGVRMRQWGKWVSEIREPKKKSRIWLGTFSTAEMA

ARAHDVAALAIKGGSAHLNFPELAYHLPRPASADPKDIQAAAAAAAAAVAIDMDVETSSPSPSPTVTETSSPAMIALSDD

AFSDLPDLLLNVNHNIDGFWDSFPYEEPFLSQSY

>AT1G77200.1

MTESSIISVKQSSPVPEEEDHHHHQQDSHRTNTKKRVRSDPGYRGVRMRTWGKWVSEIREPRKKSRIWLGTFSTPEMAAR

AHDAAALTIKGTSAVLNFPELATYLPRPASSSPRDVQAAAAVAAAMDFSPSSSSLVVSDPTTVIAPAETQLSSSSYSTCT

SSSLSPSSEEAASTAEELSEIVELPSLETSYDESLSEFVYVDSAYPPSSPWYINNCYSFYYHSDENGISMAEPFDSSNFG

PLFP

>AT5G25810.1

MIASESTKSWEASAVRQENEEEKKKPVKDSGKHPVYRGVRKRNWGKWVSEIREPRKKSRIWLGTFPSPEMAARAHDVAAL

SIKGASAILNFPDLAGSFPRPSSLSPRDIQVAALKAAHMETSQSFSSSSSLTFSSSQSSSSLESLVSSSATGSEELGEIV

ELPSLGSSYDGLTQLGNEFIFSDSADLWPYPPQWSEGDYQMIPASLSQDWDLQGLYNY

>AT5G11590.1

MAEEYYSLRSERVTQLLVPNSESDSVSDKSKAEQSEKKTKRGRDSGKHPVYRGVRMRNWGKWVSEIREPRKKSRIWLGTF

PTPEMAARAHDVAALSIKGTAAILNFPELADSFPRPVSLSPRDIQTAALKAAHMEPTTSFSSSTSSSSSLSSTSSLESLV

LVMDLSRTESEELGEIVELPSLGASYDVDSANLGNEFVFYDSVDYCLYPPPWGQSSEDNYGHGISPNFGHGLSWDL

>AT1G77640.1

MVKQELKIQVTTSSSSLSHSSSSSSSSTSALRHQSCKNKIKKYKGVRMRSWGSWVTEIRAPNQKTRIWLGSYSTAEAAAR

AYDAALLCLKGPKANLNFPNITTTSPFLMNIDEKTLLSPKSIQKVAAQAANSSSDHFTPPSDENDHDHDDGLDHHPSASS

SAASSPPDDDHHNDDDGDLVSLMESFVDYNEHVSLMDPSLYEFGHNEIFFTNGDPFDYSPQLHSSEATMDDFYDDVDIPL

WSFS

>AT1G21910.1

MVKQERKIQTSSTKKEMPLSSSPSSSSSSSSSSSSSSCKNKNKKSKIKKYKGVRMRSWGSWVSEIRAPNQKTRIWLGSYS

TAEAAARAYDVALLCLKGPQANLNFPTSSSSHHLLDNLLDENTLLSPKSIQRVAAQAANSFNHFAPTSSAVSSPSDHDHH

HDDGMQSLMGSFVDNHVSLMDSTSSWYDDHNGMFLFDNGAPFNYSPQLNSTTMLDEYFYEDADIPLWSFN

>AT1G44830.1

MVKTLQKTPKRMSSPSSSSSSSSSTSSSSIRMKKYKGVRMRSWGSWVSEIRAPNQKTRIWLGSYSTAEAAARAYDAALLC

LKGSSANNLNFPEISTSLYHIINNGDNNNDMSPKSIQRVAAAAAAANTDPSSSSVSTSSPLLSSPSEDLYDVVSMSQYDQ

QVSLSESSSWYNCFDGDDQFMFINGVSAPYLTTSLSDDFFEEGDIRLWNFC

>AT1G71520.1

MDSRDTGETDQSKYKGIRRRKWGKWVSEIRVPGTRQRLWLGSFSTAEGAAVAHDVAFYCLHRPSSLDDESFNFPHLLTTS

LASNISPKSIQKAASDAGMAVDAGFHGAVSGSGGCEERSSMANMEEEDKLSISVYDYLEDDLV

>AT1G22810.1

MDYRESTGESQSKYKGIRRRKWGKWVSEIRVPGTRDRLWLGSFSTAEGAAVAHDVAFFCLHQPDSLESLNFPHLLNPSLV

SRTSPRSIQQAASNAGMAIDAGIVHSTSVNSGCGDTTTYYENGADQVEPLNISVYDYLGGHDHV

>AT1G46768.1

MEREQEESTMRKRRQPPQEEVPNHVATRKPYRGIRRRKWGKWVAEIREPNKRSRLWLGSYTTDIAAARAYDVAVFYLRGP

SARLNFPDLLLQEEDHLSAATTADMPAALIREKAAEVGARVDALLASAAPSMAHSTPPVIKPDLNQIPESGDI

>AT3G50260.1

MDAGVAVKADVAVKMKRERPFKGIRMRKWGKWVAEIREPNKRSRLWLGSYSTPEAAARAYDTAVFYLRGPTATLNFPELL

PCTSAEDMSAATIRKKATEVGAQVDAIGATVVQNNKRRRVFSQKRDFGGGLLELVDLNKLPDPENLDDDLVGK

>AT5G67190.1

MEGGGVADVAVPGTRKRDRPYKGIRMRKWGKWVAEIREPNKRSRLWLGSYSTPEAAARAYDTAVFYLRGPTARLNFPELL

PGEKFSDEDMSAATIRKKATEVGAQVDALGTAVQNNRHRVFGQNRDSDVDNKNFHRNYQNGEREEEEEDEDDKRLRSGGR

LLDRVDLNKLPDPESSDEEWESKH

>AT4G06746.1

MVIQYKRKQEFPMVKEGMVMTEKPKRNLISSNEKRYKGIRMRKWGKWVAEIREPNKRSRIWLGSYKTAVAAARAYDTAVF

YLRGPSARLNFPEEVFKDGNGGEGLGGDMSPTLIRKKAAEVGARVDAELRLENRMVENLDMNKLPEAYGL

>AT4G36900.1

METATEVATVVSTPAVTVAAVATRKRDKPYKGIRMRKWGKWVAEIREPNKRSRIWLGSYSTPEAAARAYDTAVFYLRGPS

ARLNFPELLAGVTVTGGGGGGVNGGGDMSAAYIRRKAAEVGAQVDALEAAGAGGNRHHHHHQHQRGNHDYVDNHSDYRIN

DDLMECSSKEGFKRCNGSLERVDLNKLPDPETSDDD

>AT2G23340.1

METEAAVTATVTAATMGIGTRKRDLKPYKGIRMRKWGKWVAEIREPNKRSRIWLGSYATPEAAARAYDTAVFYLRGPSAR

LNFPELLAGLTVSNGGGRGGDLSAAYIRRKAAEVGAQVDALGATVVVNTGGENRGDYEKIENCRKSGNGSLERVDLNKLP

DPENSDGDDDECVKRR

>AT1G74930.1

MVKQAMKEEEKKRNTAMQSKYKGVRKRKWGKWVSEIRLPHSRERIWLGSYDTPEKAARAFDAAQFCLRGGDANFNFPNNP

PSISVEKSLTPPEIQEAAARFANTFQDIVKGEEESGLVPGSEIRPESPSTSASVATSTVDYDFSFLDLLPMNFGFDSFSD

DFSGFSGGDRFTEILPIEDYGGESLLDESLILWDF

>AT1G19210.1

MEGSSSSMQSKYKGVRKRKWGKWVSEIRLPNSRERIWLGSYDTPEKAARAFDAALYCLRGNNAKFNFPDNPPVISGGRNL

SRSEIREAAARFANSAEDDSSGGAGYEIRQESASTSMDVDSEFLSMLPTVGSGNFASEFGLFPGFDDFSDEYSGDRFREQ

LSPTQDYYQLGEETYADGSMFLWNF

>AT5G21960.1

MDASPKYTGVRKRKWGKWVAEIRLPNSRDRIWLGSFDSAEKAARAFDAALYCLRGPGARFNFPDNPPEIPGGRSLTPQQI

QVVASRFACEEELLPPEQHHPSPPRGDHNTEEEVIISARGEINSGSGGPTLGQVGEDNNNEGNSNDTSSYWPLIWEEENF

VGPPNSDHEFGFFTDDSTNLYFPTQQQQQHQLSSDFYYDGACEDDFSHYNINLWNF

>AT4G31060.1

MPPSPPKSPFISSSLKGAHEDRKFKCYRGVRKRSWGKWVSEIRVPKTGRRIWLGSYDAPEKAARAYDAALFCIRGEKGVY

NFPTDKKPQLPEGSVRPLSKLDIQTIATNYASSVVHVPSHATTLPATTQVPSEVPASSDVSASTEITEMVDEYYLPTDAT

AESIFSVEDLQLDSFLMMDIDWINNLI

>AT5G18450.1

MEEEQPPAKKRNMGRSRKGCMKGKGGPENATCTFRGVRQRTWGKWVAEIREPNRGTRLWLGTFNTSVEAAMAYDEAAKKL

YGHEAKLNLVHPQQQQQVVVNRNLSFSGHGSGSWAYNKKLDMVHGLDLGLGQASCSRGSCSERSSFLQEDDDHSHNRCSS

SSGSNLCWLLPKQSDSQDQETVNATTSYGGEGGGGSTLTFSTNLKPKNLMSQNYGLYNGAWSRFLVGQEKKTEHDVSSSC

GSSDNKESMLVPSCGGERMHRPELEERTGYLEMDDLLEIDDLGLLIGKNGDFKNWCCEEFQHPWNWF

>AT1G75490.1

MSSIEPKVMMVGANKKQRTVQASSRKGCMRGKGGPDNASCTYKGVRQRTWGKWVAEIREPNRGARLWLGTFDTSREAALA

YDSAARKLYGPEAHLNLPESLRSYPKTASSPASQTTPSSNTGGKSSSDSESPCSSNEMSSCGRVTEEISWEHINVDLPVM

DDSSIWEEATMSLGFPWVHEGDNDISRFDTCISGGYSNWDSFHSPL

>AT2G40340.1

MPSEIVDRKRKSRGTRDVAEILRQWREYNEQIEAESCIDGGGPKSIRKPPPKGSRKGCMKGKGGPENGICDYRGVRQRRW

GKWVAEIREPDGGARLWLGTFSSSYEAALAYDEAAKAIYGQSARLNLPEITNRSSSTAATATVSGSVTAFSDESEVCARE

DTNASSGFGQVKLEDCSDEYVLLDSSQCIKEELKGKEEVREEHNLAVGFGIGQDSKRETLDAWLMGNGNEQEPLEFGVDE

TFDINELLGILNDNNVSGQETMQYQVDRHPNFSYQTQFPNSNLLGSLNPMEIAQPGVDYGCPYVQPSDMENYGIDLDHRR

FNDLDIQDLDFGGDKDVHGST

>AT2G40350.1

MPRKRKSRGTRDVAEILRKWREYNEQTEADSCIDGGGSKPIRKAPPKRSRKGCMKGKGGPENGICDYTGVRQRTWGKWVA

EIREPGRGAKLWLGTFSSSYEAALAYDEASKAIYGQSARLNLPLLPLCQARLLHFLMNLKFVHVRIQMQDLVLVRSD

>AT3G57600.1

MEKSSSMKQWKKGPARGKGGPQNALCQYRGVRQRTWGKWVAEIREPKKRARLWLGSFATAEEAAMAYDEAALKLYGHDAY

LNLPHLQRNTRPSLSNSQRFKWVPSRKFISMFPSCGMLNVNAQPSVHIIQQRLEELKKTGLLSQSYSSSSSSTESKTNTS

FLDEKTSKGETDNMFEGGDQKKPEIDLTEFLQQLGILKDENEAEPSEVAECHSPPPWNEQEETGSPFRTENFSWDTLIEM

PRSETTTMQFDSSNFGSYDFEDDVSFPSIWDYYGSLD

>AT2G38340.1

MEKEDNGSKQSSSASVVSSRRRRRVVEPVEATLQRWEEEGLARARRVQAKGSKKGCMRGKGGPENPVCRFRGVRQRVWGK

WVAEIREPVSHRGANSSRSKRLWLGTFATAAEAALAYDRAASVMYGPYARLNFPEDLGGGRKKDEEAESSGGYWLETNKA

GNGVIETEGGKDYVVYNEDAIELGHDKTQNPMTDNEIVNPAVKSEEGYSYDRFKLDNGLLYNEPQSSSYHQGGGFDSYFE

YFRF

>AT3G11020.1

MAVYEQTGTEQPKKRKSRARAGGLTVADRLKKWKEYNEIVEASAVKEGEKPKRKVPAKGSKKGCMKGKGGPDNSHCSFRG

VRQRIWGKWVAEIREPKIGTRLWLGTFPTAEKAASAYDEAATAMYGSLARLNFPQSVGSEFTSTSSQSEVCTVENKAVVC

GDVCVKHEDTDCESNPFSQILDVREESCGTRPDSCTVGHQDMNSSLNYDLLLEFEQQYWGQVLQEKEKPKQEEEEIQQQQ

QEQQQQQLQPDLLTVADYGWPWSNDIVNDQTSWDPNECFDINELLGDLNEPGPHQSQDQNHVNSGSYDLHPLHLEPHDGH

EFNGLSSLDI

>AT5G05410.1

MAVYDQSGDRNRTQIDTSRKRKSRSRGDGTTVAERLKRWKEYNETVEEVSTKKRKVPAKGSKKGCMKGKGGPENSRCSFR

GVRQRIWGKWVAEIREPNRGSRLWLGTFPTAQEAASAYDEAAKAMYGPLARLNFPRSDASEVTSTSSQSEVCTVETPGCV

HVKTEDPDCESKPFSGGVEPMYCLENGAEEMKRGVKADKHWLSEFEHNYWSDILKEKEKQKEQGIVETCQQQQQDSLSVA

DYGWPNDVDQSHLDSSDMFDVDELLRDLNGDDVFAGLNQDRYPGNSVANGSYRPESQQSGFDPLQSLNYGIPPFQLEGKD

GNGFFDDLSYLDLEN

>AT2G40220.1

MDPLASQHQHNHLEDNNQTLTHNNPQSDSTTDSSTSSAQRKRKGKGGPDNSKFRYRGVRQRSWGKWVAEIREPRKRTRKW

LGTFATAEDAARAYDRAAVYLYGSRAQLNLTPSSPSSVSSSSSSVSAASSPSTSSSSTQTLRPLLPRPAAATVGGGANFG

PYGIPFNNNIFLNGGTSMLCPSYGFFPQQQQQQNQMVQMGQFQHQQYQNLHSNTNNNKISDIELTDVPVTNSTSFHHEVA

LGQEQGGSGCNNNSSMEDLNSLAGSVGSSLSITHPPPLVDPVCSMGLDPGYMVGDGSSTIWPFGGEEEYSHNWGSIWDFI

DPILGEFY

>AT1G64380.1

MEESNDIFQNNFSPKISEIRASLSQIILAGGPNTLDSIFSLLTPSSVESATTSFNTHNPPPPPQLGSSVYLRQRDIIEKF

HLQNRAISTPHPPLFSSTYDHHQTSELMLQAAAGSPAAAFAAALAAGRVTKKKKLYRGVRQRHWGKWVAEIRLPQNRMRV

WLGTYDTAEAAAYAYDRAAYKLRGEYARLNFPNLKDPSELLGLGDSSKLIALKNAVDGKIQSICQRVRKERAKKSVKVSK

NSSATADSSCLSSPEILSSSPVTTTTTAVTSEDSYWVSPMGLCNSENSSPVSVSVPSEVPATAEEEAMMGVDTDGFLLAR

MPSFDPELIWEVLAN

>AT4G13620.1

MITPIHTQHSLILVYINIYSPPILSKLRTGFILWTNTQKTNKKRNMEDQFPKIETSFMHDKLLSSGIYGFLSSSTPPQLL

GVPIFLEGMKSPLLPASSTPSYFVSPHDHELTSSIHPSPVASVPWNFLESFPQSQHPDHHPSKPPNLTLFLKEPKLLELS

QSESNMSPYHKYIPNSFYQSDQNRNEWVEINKTLTNYPSKGFGNYWLSTTKTQPMKSKTRKVVQTTTPTKLYRGVRQRHW

GKWVAEIRLPRNRTRVWLGTFETAEQAAMAYDTAAYILRGEFAHLNFPDLKHQLKSGSLRCMIASLLESKIQQISSSQVS

NSPSPPPPKVGTPEQKNHHMKMESGEDVMMKKQKSHKEVMEGDGVQLSRMPSLDMDLIWDALSFPHSS

>AT5G65130.1

MALNMNAYVDEFMEALEPFMKVTSSSSTSNSSNPKPLTPNFIPNNDQVLPVSNQTGPIGLNQLTPTQILQIQTELHLRQN

QSRRRAGSHLLTAKPTSMKKIDVATKPVKLYRGVRQRQWGKWVAEIRLPKNRTRLWLGTFETAQEAALAYDQAAHKIRGD

NARLNFPDIVRQGHYKQILSPSINAKIESICNSSDLPLPQIEKQNKTEEVLSGFSKPEKEPEFGEIYGCGYSGSSPESDI

TLLDFSSDCVKEDESFLMGLHKYPSLEIDWDAIEKLF

>AT2G22200.1

METASLSFPVPNTSFGVNKSMPLGLNQLTPYQIHQIQNQLNHRRSTISNLSPNRIRMKNLTPSTSKTKNLYRGVRQRHWG

KWVAEIRLPKNRTRLWLGTFETAEKAALAYDQAAFQLRGDIAKLNFPNLIHEDMNPLPSSVDTKLQAICKSLRKTEEICS

VSDQTKEYSVYSVSDKTELFLPKAELFLPKREHLETNELSNESPRSDETSLLDESQAEYSSSDKTFLDFSDTEFEEIGSF

GLRKFPSVEIDWDAISKLANS

>AT4G28140.1

MDFDEELNLCITKGKNVDHSFGGEASSTSPRSMKKMKSPSRPKPYFQSSSSPYSLEAFPFSLDPTLQNQQQQLGSYVPVL

EQRQDPTMQGQKQMISFSPQQQQQQQQYMAQYWSDTLNLSPRGRMMMMMSQEAVQPYIATKLYRGVRQRQWGKWVAEIRK

PRSRARLWLGTFDTAEEAAMAYDRQAFKLRGHSATLNFPEHFVNKESELHDSNSSDQKEPETPQPSEVNLESKELPVIDV

GREEGMAEAWYNAITSGWGPESPLWDDLDSSHQFSSESSSSSPLSCPMRPFF

>AT2G20880.1

MATAKNKGKSIRVLGTSEAEKKDEMELEEEFQFSSGKYKDSGPGSDMWLGDASSTSPRSLRKTRTFDRHNPYLVSSYATP

QPPTTTTCSVSFPFYLPPAIQNQQRFLHPNDPSGQRQQQMISFDPQQQVQPYVAQQQQQQQHLLQYWRDILKLSPSGRMM

MMNMLRQESDLPLTRPPVQPFSATKLYRGVRQRHWGKWVAEIRKPRNRTRLWLGTFDTAEEAAMAYDREAFKLRGETARL

NFPELFLNKQEPTPVHQKQCETGTTSEDSSRRGEDDSSTALAVGGVSEETGWAEAWFNAIPEEWGPGSPLWDDYHFPISN

HKDDLDATQNSSSDTI

>AT1G36060.1

MADLFGGGHGGELMEALQPFYKSASTSASNPAFASSNDAFASAPNDLFSSSSYYNPHASLFPSHSTTSYPDIYSGSMTYP

SSFGSDLQQPENYQSQFHYQNTITYTHQDNNTCMLNFIEPSQPGFMTQPGPSSGSVSKPAKLYRGVRQRHWGKWVAEIRL

PRNRTRLWLGTFDTAEEAALAYDRAAFKLRGDSARLNFPALRYQTGSSPSDTGEYGPIQAAVDAKLEAILAEPKNQPGKT

ERTSRKRAKAAASSAEQPSAPQQHSGSGESDGSGSPTSDVMVQEMCQEPEMPWNENFMLGKCPSYEIDWASILS

>AT4G39780.1

MAAIDMFNSNTDPFQEELMKALQPYTTNTDSSSPTYSNTVFGFNQTTSLGLNQLTPYQIHQIQNQLNQRRNIISPNLAPK

PVPMKNMTAQKLYRGVRQRHWGKWVAEIRLPKNRTRLWLGTFDTAEEAAMAYDLAAYKLRGEFARLNFPQFRHEDGYYGG

GSCFNPLHSSVDAKLQEICQSLRKTEDIDLPCSETELFPPKTEYQESEYGFLRSDENSFSDESHVESSSPESGITTFLDF

SDSGFDEIGSFGLEKFPSVEIDWDAISKLSES

>AT1G78080.1

MAAAMNLYTCSRSFQDSGGELMDALVPFIKSVSDSPSSSSAASASAFLHPSAFSLPPLPGYYPDSTFLTQPFSYGSDLQQ

TGSLIGLNNLSSSQIHQIQSQIHHPLPPTHHNNNNSFSNLLSPKPLLMKQSGVAGSCFAYGSGVPSKPTKLYRGVRQRHW

GKWVAEIRLPRNRTRLWLGTFDTAEEAALAYDKAAYKLRGDFARLNFPNLRHNGSHIGGDFGEYKPLHSSVDAKLEAICK

SMAETQKQDKSTKSSKKREKKVSSPDLSEKVKAEENSVSIGGSPPVTEFEESTAGSSPLSDLTFADPEEPPQWNETFSLE

KYPSYEIDWDSILA

>AT1G22190.1

MTTSMDFYSNKTFQQSDPFGGELMEALLPFIKSPSNDSSAFAFSLPAPISYGSDLHSFSHHLSPKPVSMKQTGTSAAKPT

KLYRGVRQRHWGKWVAEIRLPRNRTRLWLGTFDTAEEAALAYDKAAYKLRGDFARLNFPDLRHNDEYQPLQSSVDAKLEA

ICQNLAETTQKQVRSTKKSSSRKRSSTVAVKLPEEDYSSAGSSPLLTESYGSGGSSSPLSELTFGDTEEEIQPPWNENAL

EKYPSYEIDWDSILQCSSLVN
